# Simulations of Operation Dynamics of Different Type GaN Particle Sensors

**DOI:** 10.3390/s150305429

**Published:** 2015-03-05

**Authors:** Eugenijus Gaubas, Tomas Ceponis, Vidas Kalesinskas, Jevgenij Pavlov, Juozas Vysniauskas

**Affiliations:** Institute of Applied Research and Faculty of Physics, Vilnius University, Sauletekio av. 9-III, LT-10222 Vilnius, Lithuania; E-Mails: tomas.ceponis@ff.vu.lt (T.C.); vidas.kalesinskas@ff.vu.lt (V.K.); jevgenij.pavlov@tmi.vu.lt (J.P.); juozas.vysniauskas@ff.vu.lt (J.V.)

**Keywords:** GaN, multiplication, impact ionization, carrier lifetime

## Abstract

The operation dynamics of the capacitor-type and PIN diode type detectors based on GaN have been simulated using the dynamic and drift-diffusion models. The drift-diffusion current simulations have been implemented by employing the software package Synopsys TCAD Sentaurus. The monopolar and bipolar drift regimes have been analyzed by using dynamic models based on the Shockley-Ramo theorem. The carrier multiplication processes determined by impact ionization have been considered in order to compensate carrier lifetime reduction due to introduction of radiation defects into GaN detector material.

## 1. Introduction

Interpretation of transient currents in particle and photodetectors, due to drift of the injected charges, is a sophisticated issue which depends on many factors. The analysis of transient currents [1,2,3,4,5,6,7] due to moving charges is commonly based on the Shockley-Ramo theorem [8,9]. Alternatively, current density changes dependent on applied voltage can be simulated based on a system of Boltzmann equation, e.g., [10], using conservation laws for carrier concentration, momentum and energy. However, the latter approach is acceptable for simulation of quasi-steady-state regime, and, as emphasized in [11], “an analysis based on the Boltzmann equation with a rigorous treatment of the collision integral is prohibitive” in the dynamic theories concerned with the transport properties under dynamic conditions, *i.e.*, when the electric field varies in time and space. Therefore, the dynamic models including electric field variations are needed for deeper understanding of the transient currents in particle and photo-detectors.

Radiation-induced defects are one of the most significant limiting factors for the operational characteristics of particle detectors during high fluence irradiations. Therefore radiation harder materials such as GaN [12,13,14] and diamond [15,16] are considered as promising candidates for the design of the radiation tolerant detectors operational in the harsh environment of the future high brightness hadron collider experiments. GaN crystalline material of a proper quality is usually obtained by the MOCVD technique, and the as-grown epi-layers are rather thin. It has been evaluated [13,14] that carrier pair generation by high energy protons comprises efficiency of 40–80 pairs per μm length of the device active width per proton. The internal gain implemented through multiplication processes is also a good alternative to compensate for device performance degradation caused by radiation defects and reduction of carrier lifetime. Additionally, the thermal noise current is considerably reduced in wide band-gap materials.

Several attempts to generalize a simple Ramo’s approach to multi-electrode and arbitrary space-charge field distribution systems had been made [1,2,3,4,5,6,7]. An additional kinetic equation should be considered to determine the instantaneous velocity, as a motion velocity of charge is only postulated in Shockley-Ramo’s derivations. However, in most applications of Ramo’s theorem, the consideration of motion velocity, which should incorporate the induction (image) charge field, is ignored. Ignoring of the image charge field renders the Ramo’s current expression not practically applicable for analysis of detector signals. As usual, a current pulse is detected, which is determined secondary by carrier pairs generated in the dielectric material by a primary high energy particle. The motion velocity of these carriers changes during their drift, and it is determined by the changes of charge on the electrode. The charge, supplied to electrodes [9] by an external source (circuit) actually serves as an image charge for the moving charge within an inter-electrode space. Thereby, the superposition and redistribution of the acting electric fields should be analyzed. The field inhomogeneity effects on boundary of electrode of a fixed dimension can also be important, especially for small drifting charge. Then, the current pulse shape and duration are directly dependent on variation in time of an instantaneous position of the injected charge domain within the inter-electrode space. More complications appear within the consideration of a kinetic equation, when the injected charge can vary due to charge traps inside the inter-electrode space. These traps in dielectric material can be a reason for charge localization and local charge generation.

In more general approach, the results of Ramo’s derivation can be directly obtained from the principles of the simultaneous conservation of electrostatic energy, of charge and of charge momentum for the complete local circuit (conservative system) with fixed (location and geometry) electrodes. Conservation of electrostatic energy σ*U* = *−q*Φ requires that changes of the injected charge *q* and potential Φ attributed to this *q* should exactly be balanced by changes of the external source voltage *U* and the induced charge σ on electrode. This energy conservation is enclosed within Green’s theorem, exploited by Ramo. For a parallel-plate capacitor with inter-electrode spacing *d* in steady-state, this leads to σ = −*q*Φ/*U* = −*q*(*d*/*U*)(Φ/*d*) and to equality of acting fields *E_U_* = *U*/*d* = *E*_Φ_ = Φ/*d*, if charges on electrodes are imaging each other (σ = −*q*). The latter assumption |σ| = |*q*| is also equivalent to the charge conservation principle. On the other hand, σ = −*q* can be understood as the electrostatic induction of charge. For unit potentials accepted in [8,9], this leads to a weighting field representation *E*_Φ_ = 1/*d*. This is valid for the same space dimensionality (congruence) definition of fields, and it also represents a principal of reciprocity in electrostatics [9]. The equality of fields is essential for the evaluation of the consistent drift velocity. For drifting charge *q*, its coordinate 0 < *X_q_* < *d* changes in time *t*. To keep energy conservation, the external source should change δσ(*t*) = −δ[*q*Φ(*X_q_*(*t*))/*U*(*X_q_*(*t*))] the supplied charge to electrode, if drifting charge *q* and *U* of external source are invariable. The acting voltage *U*(*X_q_*(*t*)) changes due to voltage sharing: *U*(*X_q_*(*t*)) *=∫_Xq_^d^*(*U*/*d*)*dX* = *U*[1 − (*X_q_*/*d*)]. Changes of charge on electrode leads to the current transient *i*(*t*) = *d*σ(*t*)/*dt* within the external loop of the circuit. These current changes in time are strictly correlated with instantaneous position *X_q_*(*t*) of drifting charge and time instant. The current pulses of durations in the range from a few picoseconds to tens of microseconds with current values from a μA to tens of mA are usually measured and analyzed. In the electrostatic approach, no retardation effects are included, *i.e.*, field induction (displacement) is instantaneous, as assumed in [8,9]. The current *i*(*t*) is thereby related to a drift velocity *v_dr_*(*t*) *=* ∂(*X_q_*(*t*))/∂*t* as: *i*(*t*) = −*q*[∂(Φ(*X_q_*(*t*)))/∂*t*][*d*/*U*][1/*d*]*v_dr_*(*t*). Accepting **E**_Φ_ = −gradΦ and *E_U_ = E*_Φ_, the Ramo’s current expression is immediately obtained as *i*(*t*) = −*q*(1*/d*)*v_dr_*(*t*)*.* In the dimensionless coordinate system 0 ≤ ψ*_q_* = *X_q_*/*d* ≤ 1, this leads to the charge momentum conservation *i*(*t*) = *q*∂(ψ*_q_*(*t*))/∂*t.* To validate the global conservation laws, calibration relations adjusted to symmetry of the local system should be found. Therefore, a separate analysis of every definite circuit (device structure: capacitor, diode, *etc.*) and process (e.g., drift, trapping, diffusion, screening, *etc.*) should be made. Velocity fields should be obtained by consideration of the actual field distribution. Drift velocity changes depending on moving charge *q* instantaneous location *X_q_*, on charge density, on applied voltage, on possible screening effects. It should be pointed out, that both fields *E_U_* and *E*_Φ_ should be included into derivation of the drift velocity. The retardation (delay) can also appear due to elements of the external circuit (for instance, due to a load resistor connected in series which is capable to limit current). To derive these fields, different particular situations and regimes should be considered in detail.

The PIN type detectors have been analyzed in more detail in article [17]. It is assumed that the impact of a dielectric material in between of the capacitor electrodes is enclosed within a dielectric permittivity ε of the material and possible charge traps. The practical significance of such a consideration is reasoned by the application of capacitor type detectors, filled with diamond, and employed for the detection of relativistic particles [15,16]. Such detectors might be promising for operation in the harsh environment of high energy physics experiments, to enhance the radiation tolerance of detectors.

In this work, the models of current pulses appearing under the formation of a drifting domain of surface charge have been analyzed. The derived drift current expressions are coincident with a Ramo’s-type solution. This approach enables one to perform a drift velocity field analysis within an inter-electrode gap relative to charge injection position. The bipolar carrier drift transformation to a monopolar one after either electrons or holes in a dielectric material reach the external electrode is considered. The impact of the initial domain motion velocity and effects of the drift velocity saturation are described. The impact of the dynamic capacitance and load resistance in the formation of commonly registered drift current transients is highlighted. It has been shown that double peak waveform can appear within injected charge domain drift current transients even for fixed external voltages applied to a capacitor. It has been illustrated that the synchronous action of carrier drift, trapping, and diffusion can lead to a vast variety of possible current pulse waveforms. Also, a simplified one-dimensional approach can reproduce the main features of widely-used detectors, like those which contain short inter-electrode spaces or active layer widths significantly smaller than the lateral dimensions of the electrodes. The vector origin of the employed quantities of a surface charge domain, of an electric field and of charge motion velocity have always been kept in mind, while the scalar relations are mainly represented. Additionally, the charge multiplication processes and transients of detector current have been analyzed using this dynamic approach [17,18,19,20] based on the Shockley-Ramo theorem [8,9]. To simplify analysis, application of the dynamic approach is confined to the capacitor type detector based on thin MOCVD grown GaN epi-layers. For comparison with widely used steady-state approach, simulations of current transients for PIN diode have alternatively been performed by employing the drift-diffusion model implemented in the software package Synopsys TCAD Sentaurus.

## 2. Consideration of Current Transients in Capacitor Type Detectors

### 2.1. Vacuum Capacitor Type Detector

Let’s consider a vacuum capacitor [18] in the electrical circuit like the one in Ramo’s theorem derivation: with one electrode grounded and with a high potential on the other one, determined by an external voltage source of voltage *U,* which is connected in series with a load resistor *R* to register a transient current within an external circuit, as in [17,20], where a surface (of area *S*) charge domain of *N* electrons (−*eN*) is injected. Such a situation also appears, as shown in [21], when an injected charge domain moves with relativistic velocity. The charge induced on electrode of surface density (+σ *= eN*/*S*) by the moving charge domain should be balanced by a charge supplied to electrode by external battery for charge conservation if the external voltage is kept invariable and retardation effects can be ignored. This additional charge interacts with moving domain through Coulomb field. At Shockley-Ramo’s assumptions [8,9] concerning slow movement of the injected charge relative to velocity of the electric potential wave [10] propagation, the electric fields of interaction between moving and the electrode charges are equal when retardation effects can be ignored. The acting attraction field *E*(*t*, *X_q_*) on the domain of electrons is described in scalar representation by the relation:

(1) $$E\left(t,X_{q}\right)=-[(U/d)+(eN/S{\text{ε}}_{0}){\text{ψ}}_{q}(t)]$$

Here, *d* is the inter-electrode gap; *X_q_* is the instantaneous position of the drifting surface charge domain *q* = *eN*/*S*, varied in time *t*; ψ*_q_* = *X_q_*/*d* is the dimensionless position of drifting charge; ε_0_ is vacuum dielectric permittivity. A vector consideration with a unit vector **k**_x_ perpendicular to electrodes of a capacitor has also been employed in derivation of this relation, using vectors of electric displacement:

(2) **D** = −**k**_x_ ε_0_*eN*/*S*

and of steady-state field:

(3) **E***_s_* = −**k**_x_*U*/*d*

Then, scalar components of current density, ascribed to convection:

(4) $$j_{c}=\varepsilon_{0}\left(\frac{\partial E(t,X_{q})}{\partial t}\right)=-\left(\frac{-eN}{S}\right)\frac{\partial \psi_{q}(t)}{\partial t}=\left(\frac{eN}{S}\right)\frac{\partial \psi_{q}(t)}{\partial t}$$

and to displacement ones:

(5) $$j_{ds}=\varepsilon_{0}\left(\frac{\partial [-[(U/d)-(eN/S\varepsilon_{0})(1-\psi_{q}(t))]]}{\partial t}\right)=\left(\frac{eN}{S}\right)\left(\frac{\partial \psi_{q}(t)}{\partial t}\right)$$

are obtained. These expressions coincide with ones derived in Shockley-Ramo’s theory [8,9] at well-known assumption that negative charge moves against the direction of acting field. Thus, the expression for current transient can completely be described when a motion velocity:

(6) $$\text{v}(t)=d\frac{\partial {\text{ψ}}_{q}(t)}{\partial t}$$

of the injected domain is derived*.*

The domain position and motion velocity changes in time can be described by solving boundary problem ψ*_q_*(*t* = 0) = ψ*_q_*_0_ as well as ψ*_q_*(*t =* τ*_tr_*) *=* 1 for a domain transit time τ*_tr_* and by combining Newton’s dynamic equation as well as the momentum and energy conservation laws. From the Newton’s equation, the time dependent changes of the dimensionless position ψ*_q_*(*t*) are expressed as:

(7) $$\psi_{q}(t)=\frac{\sinh\sqrt{\frac{\tau_{tr}}{\tau_{M}}}\frac{t}{\tau_{tr}}}{\sinh\sqrt{\frac{\tau_{tr}}{\tau_{M}}}}+\psi_{q0}\frac{\sinh\sqrt{\frac{\tau_{tr}}{\tau_{M}}}(1-\frac{t}{\tau_{tr}})}{\sinh\sqrt{\frac{\tau_{tr}}{\tau_{M}}}}+\frac{\tau_{M}}{\tau_{TOF}}[2\frac{\sinh\frac{1}{2}\sqrt{\frac{\tau_{tr}}{\tau_{M}}}\cosh\sqrt{\frac{\tau_{tr}}{\tau_{M}}}(\frac{t}{\tau_{tr}}-\frac{1}{2})}{\sinh\sqrt{\frac{\tau_{tr}}{\tau_{M}}}}-1]$$

The transit time τ*_tr_* can be evaluated from the energy conservation relation:

(8) $$\left(\frac{Nm_{e}d^{2}}{2}\right){\left(v_{0}+{\frac{\partial \psi_{q}(t)}{\partial t}|}_{t=\tau tr}\right)}^{2}=eN\left(\left(\frac{U}{d}\right)+{\left(\frac{eN}{S\varepsilon_{0}}\right)\psi_{q}(t)|}_{t=\tau tr}\right)d$$

with initial drift velocity *v_0_*. For *v_0_* = 0, this reads:

(9) $$\tau_{tr}=\sqrt{\frac{m_{e}}{e}(2\frac{d^{2}}{U}\frac{\varepsilon_{0}Sd}{eN})/(\frac{d^{2}}{U}+\frac{\varepsilon_{0}Sd}{eN})}$$

Here, *e* is the elementary charge, *m_e_* denotes the mass of electron. Consequently, from the momentum conservation law, the mobility μ*_v_* can formally be introduced as μ*_v_* = (*e*/*m_e_*)τ*_tr_*. Then, the characteristic times of dielectric relaxation:

(10) $$\tau_{M}=\frac{\varepsilon_{0}Sd}{Ne\mu_{v}}$$

and of free flight:

(11) $$\tau_{TOF}=\frac{d^{2}}{U\mu_{v}}$$

can be defined. The τ*_M_* determines an ability of the moving charge to respond to charge/voltage variations on electrodes, while τ*_TOF_* characterizes the electrodes by the charge balance duration through convective component of displacement current, *i.e.*, via free flight of charge carriers. If retardation and magnetic field effects can be ignored (*i.e.*, for Shockley-Ramo [8,9] regime), these response times should be equal: τ*_TOF_* = τ*_M_*. This is determined by the correlated, quasi-static Coulomb interaction of moving charges and charges on electrodes. Then, velocity changes in time *v*(*t*) are evaluated from Equation (1) by *v*(*t*) = *d*(*∂ψ_q_*(*t*)/*∂t*) for 0 ≤ *t* ≤ τ*_tr_* and ψ*_q_*_0_ ≤ ψ*_q_* ≤ 1.

The dimensionless position and drift velocity increases nearly exponentially with time for Ramo’s regime. This is caused by increase of acting electric field with reduction of a gap between a moving domain and the electrode. Relation τ*_TOF_*/τ*_M_* = 1 determines the Shockley-Ramo regime which can only be implemented for a definite set of geometrical and injection/biasing parameters. For τ*_TOF_*/τ*_M_* > 1, the injected charge screens rapidly that on electrodes, and motion of this charge to electrodes is only possible through diffusion. Contrary, in the case of τ*_TOF_*/τ*_M_* < 1, the lateral changes of fields and velocities within injected domain and electrode planes should be considered, thereby this leads to retardation and magnetic field effects. The latter case can be analyzed by introduction of retarded Green’s function for potentials [10] or the relativistic representation of charge [21]. Actually, a system of inhomogeneous wave equations [10] with charge and current density sources for both scalar and vector potentials should then be solved. External circuit can also significantly modify the shape of current transient.

### 2.2. Detector Made of Capacitor Filled with Dielectric

**Figure 1:**
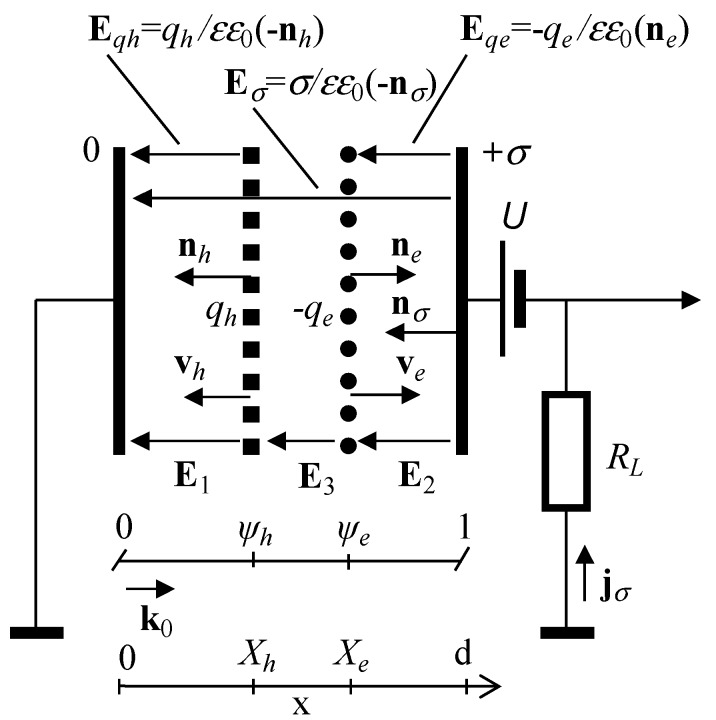
Schematics of the considered circuit. Symbols denote as follows: *X_e_*_,*h*_ is the instantaneous position of the drifting one-sided surface charge domain of the electron (–*q_e_*) or hole (*q_h_*) (with normal vectors **n***_e_* and **n***_h_*), respectively; **E**_1_ = **E***_qh_* + **E**_σ_ is the electric field caused by a superposition of the hole surface charge (*q_h_*) domain and the surface (of an area *S*) charge (+σ , with a normal vector n_σ_) on the high potential electrode; **E**_2_ = **E***_qe_* + **E**_σ_ is the electric field caused by the superposition of the surface charge (–*q_e_*) domain and the surface charge (+σ); **E**_3_ = **E**_σ_ is the field between the electron and hole domains; **v***_e_*_,*h*_ is the instantaneous velocity vector of a drift of the surface charge domain −*q_e_* or *q_h_*, respectively; *d* is the inter-electrode distance; ε and ε_0_ are the material and vacuum dielectric permittivity, respectively; **k**_0_ is the unit normal vector (necessary to represent the projections through scalar product of vectors, for the case of boundary effects) within the unidirectional coordinate system; ψ*_h_* and ψ*_e_* are the dimensionless, normalized positions of the drifting hole or electron domains, respectively; **j**_σ_ is the current density; *U* is the external source of voltage; *R_L_* is the load resistor.

As usual, a quasi-neutral domain of excess carriers is initially generated in particle detectors. It is accepted the domains of surface charge are vector quantities, characterized by the surface charge density and a normal. The sign (polarity) of the injected charge and a direction of the drift velocity vector are also included. The surface charge (**σ**, **q**) vectors, determined commonly [17,18,19,20] as a discontinuity of electrostatic displacement vectors **D**, for instance in Figure 1:

(12) −**q**_e_ = (**D**_2_ − **D**_3_) = **n_e_**εε_0_*E_qe_*

are initially considered. Then, the scalar equations for an instantaneous field distribution are analyzed. On the basis of the electrostatic calibration laws, consideration of the grounded circuit (with asymmetry caused by applied external field and motion velocity) is more efficient to get the compact equations, as made in [8,9,17,20]. The grounded circuit means the initial pre-calibration with 0 ≤ *x* ≤ *d*, where it is assumed that charges (σ) and potentials (φ) are measured relative to a ground with σ*_g_* = 0 and φ*_g_* = 0. Due to applied the external field source, the injected carriers can be separated into oppositely moving surface charge sub-domains *q_e_* and *q_h_*, which induce charges of an opposite sign on electrodes. The positive charge on the grounded electrode induced by a drifting charge domain is moved by the external source (battery) to the electrode of the high potential and vice versa. The latter charge transfer current is actually measured within the external circuit as a signal of either the charge domain drift or the charge density change. A sketch of the instantaneous field components is presented in Figure 1.

The instantaneous electric field distribution along the *x* axis (0 ≤ *x* ≤ *d*) for the bipolar drift can be expressed in the scalar representation through the surface charge densities, denoted in Figure 1, as:

(13) $$E(x)=\{\begin{array}{c} E_{1}=-\frac{\text{σ}}{{\text{εε}}_{0}}-\frac{q_{h}}{{\text{εε}}_{0}}\text{ for }x<X_{h}\\ E_{3}=-\frac{\text{σ}}{{\text{εε}}_{0}}\text{ for }X_{h}<x<X_{e}\\ E_{2}=-\frac{\text{σ}}{{\text{εε}}_{0}}-\frac{q_{e}}{{\text{εε}}_{0}}\text{ for }x>X_{e} \end{array}$$

Field discontinuity at the location of the surface charge domains is included as:

(14) $$E=\{\begin{array}{c} E_{3}(X_{h})-E_{1}(X_{h})=\frac{q_{h}}{{\text{εε}}_{0}}\text{ for }X_{h}\\ E_{2}(X_{e})-E_{3}(X_{e})=-\frac{q_{e}}{{\text{εε}}_{0}}\text{ for }X_{e} \end{array}$$

It is more convenient to consider a dimensionless, normalized quantity of charge position ψ*_e_*_,*h*_ *= X_e_*_,*h*_/*d*. Then, boundary conditions for the charge drift space should be taken as follows:

(15) $$\psi =\psi_{0}\text{ for }t=0$$

and:

(16) $$\psi =1\text{ for }t=\tau_{dr}$$

The relation between the surface charge (+σ) on the high potential electrode and the external voltage *U* is obtained by taking the second Poisson integral, and the solution for a scalar surface charge σ can be written as:

(17) $$\sigma =U\frac{\varepsilon \varepsilon_{0}}{d}-q_{e}(1-\psi_{e})-q_{h}\psi_{h}\equiv UC_{e-h}$$

Here, *C_e-h_* is the capacitance of a unit surface area of the system. The detailed solutions for different monopolar, bipolar and mixed drift regimes are presented below. It is worth noting that in the case of bipolar drift, the charge σ on the high potential electrode becomes dependent on the instantaneous location ψ*_e_*, ψ*_h_* of the separate electron and hole domains. This leads to the scalar expressions for the fields which act (including a direction of the drift velocity vector) on the electrons (*E*_2_) and holes (*E*_1_) also being dependent on the instantaneous location of the drift counter-partners as:

(18) $$E(\text{ψ})=\{\begin{array}{c} E_{1}=-\frac{U}{d}+\frac{q_{e}}{{\text{εε}}_{0}}(1-{\text{ψ}}_{e})-\frac{q_{h}}{{\text{εε}}_{0}}(1-{\text{ψ}}_{h})\text{ for }x<X_{h}\\ E_{2}=-\frac{U}{d}-\frac{q_{e}}{{\text{εε}}_{0}}{\text{ψ}}_{e}+\frac{q_{h}}{{\text{εε}}_{0}}{\text{ψ}}_{h}\text{ for }x>X_{e} \end{array}$$

External source *U* induces a positive surface charge *q_C_* = *C_g_U* on the high potential electrode, which is positioned at the distance *d* from the grounded electrode. Here, *C_g_* is the geometrical capacitance of a unit area of a capacitor plate. The grounded electrode is located at the beginning of the coordinate system (ψ = 0). Introduction of the plane surface domain of the electrons or holes, with a surface charge density *q_e_* and *q_h_* at the instantaneous position *X*_0_ causes a change in the charge on the electrode. As the electrodes are only connected to an external measurement circuit, the moving charge domains −*q_e_* and *q_h_* induce a current within the external circuit, which is determined by the surface charge δσ changes in time: *d*σ/*dt*. To find these changes, the superposition of fields (**E**_qe_ = **n**_e_(−*q_e_*/εε_0_), **E**_qh_ = −**n**_h_(+*q_h_*/εε_0_), and **E**_σ_ = −**n**_σ_(+σ/εε_0_)) induced by the surface charges *q_e_*, *q_h_* and σ (for the initial instant σ *= q_C_* ) should be considered.

#### 2.2.1. Monopolar Drift of Surface Charge Domain

**Figure 2:**
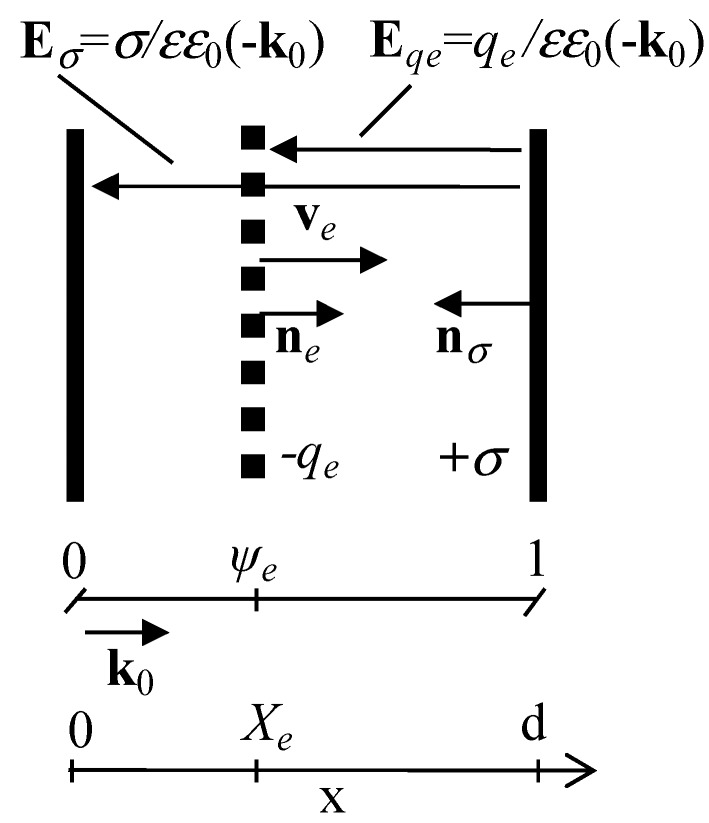
Sketch of a drifting negative surface charge domain. Symbols denote as follows: *X*_e_ the instantaneous position of the moving surface (with a normal vector **n***_e_*) charge (–*q*_e_) of the injected electron domain, **E***_qe_* the electric field caused by the surface charge *q_e_* domain, **v***_e_* the instantaneous velocity vector of charge *q*_e_ motion, *d* the inter-electrode distance, +σ surface (with a normal vector **n**_σ_) charge on the high potential electrode, **E**_σ_ the electric field caused by surface charge σ, **k**_0_ is a unit vector in the unidirectional coordinate system. The external circuit is the same as in Figure 1.

For constant values of *U* and for the induced negative surface charge domain −*q_e_*, the electric field step appears at local position *X_e_* of the injected surface charge domain, Figure 2.

In rearranging the vector equations to scalar ones for a one-dimensional problem (with surface charges *q_e_* and σ) of electric fields (*E_qe_* and *E*_σ_), and by assuming the proper signs of the equation components, matched with the surface charge normal directions (shown in Figure 2) and charge signs, one can express the instantaneous electric field distribution along the *x* axis (0 ≤ *x* ≤ *d*) as follows:

(19) $$E(x)=\left\{ \begin{array}{l} -\frac{\text{σ}}{{\text{εε}}_{0}}\text{ for }x<X_{e}\;\\ -\frac{\text{σ}}{{\text{εε}}_{0}}-\frac{q_{e}}{{\text{εε}}_{0}}\text{ for }x>X_{e} \end{array}\right.\;$$

To find the surface charge on electrode σ related to *q_e_* and *X_e_*, and to associate σ with potential *U* on the electrodes, the second Poisson integral should be taken, as:

(20) $$-U=\int_{0}^{d}E(x)dx\equiv \int_{0}^{X_{0}}E(x)dx+\int_{X_{0}}^{d}E(x)dx$$

Integration of Equation (20) gives the relation of σ with *q_e_*, *X_e_* and *U*, as:

(21) $$\sigma =U\frac{\varepsilon \varepsilon_{0}}{d}-q_{e}(1-\frac{X_{e}}{d})$$

Inserting σ from Equation (21) into Equation (19), the field distribution is finally obtained as:

(22) $$E(x)=\left\{ \begin{array}{l} E_{1}=-\frac{U}{d}+\frac{q_{e}}{{\text{εε}}_{0}}\left(1-\frac{X_{e}}{d}\right)\text{ for }x<X_{e}\\ E_{2}=-\frac{U}{d}-\frac{q_{e}}{{\text{εε}}_{0}}\frac{X_{e}}{d}\text{ for }x>X_{e} \end{array}\right.$$

It is worth noting that Equation (22) represents field distribution with calibrated charge and potential. It can be noticed from Equation (20) that fields *E*_1_ and *E*_2_ have opposite directions for a non-biased capacitor (*U* = 0). This appears due to the symmetry of the displacement vectors those define the injected surface charge *q_e_* at *X_e_*. A simple re-arrangement of the coordinate system consistent with *X_e_* yields +*q_e_X_e_*/*d*εε_0_ = +*q_e_*(1 − *X_e_*)/*d*εε_0_, —as obtained by a proper shift of the beginning of the coordinate system. The charges induced on the opposite electrodes coincide then exactly. Thereby, no current is induced within an external circuit loop if there is no asymmetry source (either external voltage or initial drift velocity).

The solution for a scalar surface charge in Equation (21) can be re-arranged as:

(23) $$\sigma =U\frac{\varepsilon \varepsilon_{0}}{d}[1-\frac{q_{e}d}{\varepsilon \varepsilon_{0}U}(1-\frac{X_{e}}{d})]\equiv UC_{Sq,e}$$

where *C_Sq_*_,*e*_ ≥ 0 is referred to as the dynamic capacitance of a system of a surface (*S*) area unit, expressed as:

(24) $$C_{Sq,e}=\frac{\varepsilon \varepsilon_{0}}{d}[1-\frac{q_{e}}{\varepsilon \varepsilon_{0}}\frac{d}{U}(1-\frac{X_{e}}{d})]$$

For invariable quantities of the external voltage *U* and the induced charge surface density *q_e_*, the current density, as a vector quantity, can be described using Equation (22) as:

(25) $$\vec{j}=\varepsilon \varepsilon_{0}\frac{d{\vec{E}}_{2}}{dt}\equiv -q_{e}\frac{1}{d}\frac{dX_{e}}{dt}{\vec{k}}_{\text{0}}=\varepsilon \varepsilon_{0}\frac{d{\vec{E}}_{\text{1}}}{dt}$$

This expression exactly coincides with the expression for Ramo’s current. It can easily be noticed that the vector of the charge domain motion velocity is opposite to the electric field vector, *i.e.*, the electron charge domain can only move towards the high potential electrode (Figure 2). Also, the drift current is exactly equal to the displacement current to complete the inter-electrode circuit.

On the other hand, a scalar description of the current density module can be routinely introduced by considering the charge changes relative to time on the basis of the system capacitance variation. For *U* = const employing Equation (23), one can obtain:

(26) $$j=\frac{d\sigma }{dt}=\frac{dC_{Sq,e}}{dt}U=q_{e}\frac{1}{d}\frac{dX_{e}}{dt}$$

The expressions derived for the current density or current, as well as the Ramo’s current [8], do not allow one to describe the current transients directly. For a description of the current transient, a concerted solution for the charge motion velocity should be obtained. To follow the electrostatic approach, the charge movement is only considered to be caused by the electrostatic field component acting between the high potential electrode and the surface charge domain. Thus, the additional equation for the velocity of the charge movement is expressed as follows:

(27) $$\frac{dX_{e}}{dt}{\vec{k}}_{\text{0}}=v(-{\vec{k}}_{\text{0}})$$

In a capacitor filled with a dielectric, any ionizing particle is able to generate secondary excess charge carriers, which actually drift within the inter-electrode gap by inducing a current transient. The electron domain drifts, after the holes are extracted directly to the grounded electrode, and if the injection of carriers by highly absorbed light or ionizing particles is implemented nearby this contact, *i.e.*, *X_e_*/*d* ≈ 0.

Then the solution for a scalar surface charge in Equation (21) can be re-arranged as:

(28) $$\sigma =U\frac{\varepsilon \varepsilon_{0}}{d}[1-\frac{\tau_{TOF,e}}{\tau_{Mq,e}}(1-\frac{X_{e}}{d})]\equiv UC_{Sq,e}$$

Here, τ*_TOF_*_,*e*_ is the time of flight, and τ*_Mq_*_,*e*_ is the dielectric relaxation time of the induced charge domain, introduced by equations similar to Equations (11) and (10). Equation (28) implies that the dynamic capacitance *C_Sq_*_,*e*_ of the system depends on the instantaneous location of the surface charge domain within the inter-electrode space, and it changes with the motion of this domain. However, it depends on the system relaxation characteristic times (from another point of view, on the induced space charge and applied voltage) to maintain the balance of the electrostatic fields and energy. The capacitance of the system decreases due to an injected charge *q_e_*, relative to its value without an induced charge. This is caused by a reduction of the surface charge on electrode σ, which is involved in the termination of *q_e_*. However, the requirement for non-negative *C_Sq_*_,*e*_ ≥ 0 values leads to a limited density of surface charge *q_e_* which can be moved.

##### Drift of Electron Domain with Zero Initial Velocity

For the description of a current transient, a concerted solution for the charge motion velocity should be obtained. To follow the electrostatic approach, the charge movement is only considered to be caused by the electrostatic field component acting between the high potential electrode and the surface charge domain, if the initial velocity is zero. Thus, the additional equation for the velocity of charge movement is expressed as follows:

(29) $$\frac{dX_{e}}{dt}{\vec{k}}_{\text{0}}=\mu_{e}E(X_{e})(-{\vec{k}}_{\text{0}})$$

It is more convenient to consider the dimensionless, normalized quantity of ψ = *X*_e_/*d* for the charge domain position. Then, Equation (29) is rearranged by inserting the *E*_2_(ψ) expression taken from Equation (22) and maintaining the signs obtained in the vector relation (Equation (29)) as well as the characteristic times (Equations (10) and (11)), and can be written as:

(30) $$\frac{d\psi_{e}}{dt}=\frac{1}{\tau_{TOF,e}}[1+\frac{\tau_{TOF,e}}{\tau_{Mq,e}}\psi_{e}]=\frac{1}{\tau_{Mq,e}}[\frac{\tau_{Mq,e}}{\tau_{TOF,e}}+\psi_{e}]$$

The boundary conditions for this kinetic equation should be taken as follows:

(31) $$\;\psi_{e}=\psi_{0}\text{ for }t=0$$

and:

(32) $$\;\psi_{e}=1\text{ for }t=t_{dr,e}$$

Actually, Equations (31) and (32) only make sense for consideration of the vertex of the induced charge current pulse determined, because the charge domain only moves from the initial injection position to the electrode where it is annihilated. Thus, the solution of Equation (30) with the boundary conditions represented by Equations (31) and (32), is only determined for the time (*t*) interval 0 ≤ *t* ≤ *t_dr_*. It is obtained by integrating Equation (30) as:

(33) $$\psi_{e}(t)=\psi_{0}e^{\frac{t}{\tau_{Mq,e}}}+\frac{\tau_{Mq,e}}{\tau_{TOF,e}}(e^{\frac{t}{\tau_{Mq,e}}}-1)$$

By inserting the boundary condition Equation (32) into Equation (33), the drift time *t_dr_* is evaluated as:

(34) $$t_{dr,e}=\tau_{Mq,e}\ln\frac{\frac{\tau_{Mq,e}}{\tau_{TOF,e}}+1}{\frac{\tau_{Mq,e}}{\tau_{TOF,e}}+\psi_{0}}$$

The pulse vertex of the induced charge drift current transient, *i.e.*, the *j*(*t*) function, can consequently be described (by rearranging Equation (26) as; *j* = *q_e_d*ψ*_e_*/*dt* where *d*ψ*_e_*/*dt* is expressed by using the right hand side expression of Equation (30) with ψ_e_(*t*) from Equation (33)) as:

(35) $$j(t)=\frac{q_{e}}{\tau_{Mq,e}}e^{\frac{t}{\tau_{Mq,e}}}(\psi_{0}+\frac{\tau_{Mq,e}}{\tau_{TOF,e}})$$

Equation (35) implies that the prevailing drift of an electron domain is featured by a non-decreasing (increasing or nearly invariable) vertex current within the current transient. The published (e.g., [2,4]) negative exponents in the description of current transients should hint that other (respectively to drift) charge dissipation (namely, carrier capture) processes dominate. As it can be easily seen, the same expression is obtained using Equation (24), when considering changes of the surface charge on the electrode due to time dependent variations of the system capacitance.

Equations (33) and (34) generally show an exponential increase (with time) in the domain position (and consequently of current pulse vertex) during its drift. This increase can be explained by the necessity to balance changes of the surface charge σ with capacitance *C_Sq_*_,*e*_ variations, or by a voltage drop (which accelerates *q_e_*) reduction ~(1 − ψ*_e_*(*t*)) with the approach of ψ*_e_*(*t*) to its boundary value ψ*_e_* = 1. The drift of the injected domain is generally determined by the superposition of acting fields.

##### Drift of an Electron Domain with a Finite Initial Velocity

The problem of negative charge domain drift (starting at ψ_0_ with a finite initial movement velocity *v*_0_) appears when a monopolar drift of a domain within an inter-electrode space is considered after one of the bipolar drift counter-partners (for instance, of holes) disappears at the electrode. To describe the drift current transients in a dielectric or semiconductor filled capacitor, the solution of a kinetic equation can be sufficient. The kinetic equation for the case of a finite initial velocity *v*_0*e*_ is rearranged as:

(36) $$\frac{d\psi_{e}}{dt}=\frac{v_{0e}}{d}+\frac{1}{\tau_{Mq,e}}[\frac{\tau_{Mq,e}}{\tau_{TOF,e}}+\psi_{e}]$$

Solution of this equation (equivalent to Equation (30) for *v*_0*e*_ = 0) is obtained as follows:

(37) $$\psi_{e}(t)=\psi_{0e}e^{\frac{t}{\tau_{Mq,e}}}+(\frac{v_{0e}}{d}\tau_{Mq,e}+\frac{\tau_{Mq,e}}{\tau_{TOF,e}})(e^{\frac{t}{\tau_{Mq,e}}}-1)$$

with the respective drift time:

(38) $$t_{dr,e}=\tau_{Mq,e}\ln\frac{\frac{v_{0e}}{d}\tau_{Mq,e}+\frac{\tau_{Mq,e}}{\tau_{TOF,e}}+1}{\frac{v_{0e}}{d}\tau_{Mq,e}+\frac{\tau_{Mq,e}}{\tau_{TOF,e}}+\psi_{0,e}}$$

##### Impact of the Drift Velocity Saturation Effect

Incorporation of the initial velocity and velocity saturation effects is the most complicated for the case of bipolar drift. This appears to be due to the acting field dependence (for each type of particle domain) on both drift counter-partners position, varied in time. However, solution of this problem leads to very cumbersome expressions. Therefore, steps for solving this problem are only discussed below.

Carrier drift velocity in dielectric or semiconducting materials usually exhibits saturation at strong fields. Therefore, the value of drift velocity can be non-linearly dependent on an applied field in the range of strong electric fields. In the analysis of bipolar drift within capacitor inter-electrode space, a zero initial velocity is assumed as a neutral space charge domain is initially injected. The problem of initial velocity only appears after one of the bipolar drift counter-partners (for instance, holes) disappears at an electrode.

The drift velocity saturation effect can only be considered by solution of a system of equations, obtained using, for instance, equations, as:

(39A) $$\frac{d{\text{ψ}}_{h}}{dt}=\frac{{\text{μ}}_{h}}{1+\frac{{\text{μ}}_{h}|E_{h}({\text{ψ}}_{h},{\text{ψ}}_{e})|}{v_{thr,h}}}\frac{\left|E_{h}({\text{ψ}}_{h},{\text{ψ}}_{e})\right|}{d}\text{ with }|E_{h}|=|\frac{U}{d}+\frac{q_{h}}{{\text{εε}}_{\;0}}(1-{\text{ψ}}_{h})-\frac{q_{e}}{{\text{εε}}_{\;0}}(1-{\text{ψ}}_{e})|$$

(39B) $$\frac{d{\text{ψ}}_{e}}{dt}=\frac{\left(1-{\text{ψ}}_{e}\right)}{{\text{τ}}_{b}}$$

(39C) $$\frac{d{\text{ψ}}_{e}}{dt}=\frac{{\text{μ}}_{e}}{1+\frac{{\text{μ}}_{e}|E_{e}({\text{ψ}}_{h},\;{\text{ψ}}_{e})|}{v_{thr,e}}}\frac{\left|E_{e}({\text{ψ}}_{h},\;{\text{ψ}}_{e})\right|}{d}\text{ with }|E_{e}|=|\frac{U}{d}+\frac{q_{e}}{{\text{εε}}_{0}}{\text{ψ}}_{e}-\frac{q_{h}}{{\text{εε}}_{0}}{\text{ψ}}_{h}|$$

Then, by replacing (Equation (39B)) with (Equation (39C)), a quadratic equation for ψ*_e_,* relating it with ψ*_h_*, is obtained. The root ψ*_e_*(ψ*_h_*) of this quadratic equation then is inserted into Equation (39A). This leads to the *E_h_* expression, only containing constants and ψ*_h_* product components. The latter equation with its respective coefficients becomes rather cumbersome. Then, Equation (39A) can be solved. However, it leads to a transcendental equation of the type:

(40) $$\psi_{h}(t)=\psi_{0,h}+\frac{\mu_{e}|E_{thr}|}{d}t-\frac{\mu_{e}|E_{thr}|}{\mu_{h}}\int_{\psi_{0h}}^{\psi_{h}(t)}\frac{d\psi }{\left|E_{h}(\psi )\right|}$$

The ψ*_h_*(*t*) values obtained enable one to calculate *E_h_*(*t*) and ψ*_e_*(*t*) through the initial position ψ_0,*h*_ of the holes domain. Then, *E_e_*(*t*) and current density *j*(*t*) can be simulated. However, the solutions of Equation (39) and the current density transients can only be simulated by using numerical solutions. Here, the transit time is obtained using the boundary conditions.

Next, the kinetic equation for a drifting monopolar charge position change is re-arranged as:

(41) $$\frac{d\psi }{dt}=\frac{\mu_{e}(E)}{d}E=\frac{\mu_{e}}{d}E_{thr}\frac{\frac{1}{\tau_{Mq,e}}(\frac{\tau_{Mq,e}}{\tau_{TOF,e}}+\psi )}{\left[\frac{\mu_{e}}{d}E_{thr}+\frac{1}{\tau_{Mq,e}}(\frac{\tau_{Mq,e}}{\tau_{TOF,e}}+\psi )\right]}$$

Solution of Equations (39A) and (41) leads to a transcendental equation (which can be re-arranged as Lambert-W type function expressions [22]) for the domain position changes as a function of time:

(42) $$\psi (t)=\psi_{0}+\frac{\mu_{e}}{d}E_{thr}t-\frac{\mu_{e}}{d}\tau_{Mq,e}E_{thr}\ln\frac{\frac{\tau_{Mq,e}}{\tau_{TOF,e}}+\psi (t)}{\frac{\tau_{Mq,e}}{\tau_{TOF,e}}+\psi_{0}}$$

with drift time, obtained using the boundary conditions as:

(43) $$t_{tr}=\tau_{Mq,e}\ln\frac{\frac{\tau_{Mq,e}}{\tau_{TOF,e}}+1}{\frac{\tau_{Mq,e}}{\tau_{TOF,e}}+\psi_{0}}+\frac{(1-\psi_{0})d}{\mu_{e}E_{thr}}$$

The description of current transients due to monopolar drift of electrons, discussed in Section 2.2.1, is performed similarly for monopolar drift of the injected hole domain.

#### 2.2.2. Bipolar Drift of a Surface Charge Domain in a Capacitor Filled with a Dielectric

The bipolar drift leads to a current dependent only on the change of separation (*X_e_* − *X_h_*) between the sub-domains of *q_e_* and *q_h_* and the rate of position change in time of sub-domains.

In particle detectors, a quasi-neutral domain of excess carriers is usually initially generated. Then, owing to diffusion and the applied steady-state field, these carriers can be separated into oppositely moving surface charge sub-domains *q_e_* and *q_h_*, which induce charges on electrodes and determine a field between them (*q_e_* and *q_h_*), to hold the initial quasi-neutrality. A sketch of the field components is presented in Figure 1.

The instantaneous electric field distribution along the *x* axis (0 ≤ *x* ≤ *d*) and relation between a surface charge +σ on the high potential electrode and external voltage *U* is obtained by taking the second Poisson integral. These expressions are represented in Equations (13)–(18). Then, the induced current density, due to a bipolar drift, is expressed as follows:

(44) $$j=\frac{d\sigma }{dt}=-(q_{h}\frac{1}{d}\frac{dX_{h}}{dt}-q_{e}\frac{1}{d}\frac{dX_{e}}{dt})\;=q(\frac{d\psi_{h}}{dt}+\frac{d\psi_{e}}{dt})$$

It can be noticed that, owing to *q* = |*q_h_*| = |*q_e_*| and *q_h_* = −*q_e_* (*d*ψ*_h_*/*dt* < 0), the current density in Equation (44) represents a sum of Ramo’s-like [8] components. However, the drift velocities are coherent for the bipolar drift time τ*_b_* as:

(45) $$\frac{\left(1-\psi_{e}\right)}{\frac{d\psi_{e}}{dt}}=\frac{\psi_{h}}{-\frac{d\psi_{h}}{dt}}=\tau_{b}$$

Here, the drift directions are indicated by a sign at the scalar velocity. Several situations exist for the bipolar drift:

(46) $$\frac{{\text{ψ}}_{0}}{-\frac{d{\text{ψ}}_{h}}{dt}}=\frac{\left(1-{\text{ψ}}_{0}\right)}{\frac{d{\text{ψ}}_{e}}{dt}}={\text{τ}}_{b}\text{ for }{\text{τ}}_{b}={\text{τ}}_{dr,e}={\text{τ}}_{dr,h}$$

(47) $$\frac{{\text{ψ}}_{0}}{-\frac{d{\text{ψ}}_{h}}{dt}}=\frac{\left(1-{\text{ψ}}_{e}\right)}{\frac{d{\text{ψ}}_{e}}{dt}}={\text{τ}}_{dr,h}\text{ for }{\text{τ}}_{b}={\text{τ}}_{dr,h}<{\text{τ}}_{dr,e}$$

and:

(48) $$\frac{{\text{ψ}}_{h}}{-\frac{d{\text{ψ}}_{h}}{dt}}=\frac{\left(1-{\text{ψ}}_{0}\right)}{\frac{d{\text{ψ}}_{e}}{dt}}={\text{τ}}_{dr,e}\text{ for }{\text{τ}}_{b}={\text{τ}}_{dr,e}<{\text{τ}}_{dr,h}$$

The regime (Equation (46)) of the synchronous existence of both type carriers within the entire inter-electrode gap can only be realized for a single point of the charge domains injection ψ_0_. A bipolar drift can either change to a monopolar drift of an electron domain after holes reach a grounded electrode or it can become a monopolar drift of a hole domain after electrons reach the high potential electrode. These latter situations depend on the injection location ψ_0_ *=* ψ_0*h*_ = ψ_0*e*_ and on the mobility of the carriers.

##### Pure Bipolar Drift

In the case of a pure bipolar drift (Equation (46)), a system of kinetic equations and their solutions are presented as follows:

(49) $$\begin{array}{l} \frac{d\psi_{h}}{dt}=-\frac{\psi_{0}}{\tau_{b}};\;\frac{d\psi_{e}}{dt}=\frac{1-\psi_{0}}{\tau_{b}}\\ \psi_{h}=\psi_{\;0}(1-\frac{t}{\tau_{b}});\;\psi_{e}=\psi_{0}(1-\frac{t}{\tau_{b}})+\frac{t}{\tau_{b}} \end{array}$$

These solutions satisfy the boundary conditions:

(50) $$\begin{array}{l} \psi_{h}{|}_{t=0}=\psi_{\;0};\;\psi_{e}{|}_{t=0}=\psi_{0}\\ \psi_{h}{|}_{t=\tau_{b}}=0;\;\psi_{e}{|}_{t=\tau_{b}}=1 \end{array}$$

The time τ*_b_* of the bipolar drift is obtained by integrating the expression for drift velocity (using Equations (46)–(48)) as:

(51) $$\begin{array}{l} \frac{d\psi_{h}}{dt}=\frac{\mu_{h}}{d}E_{1}\;\\ \int_{\psi_{0}}^{0}d\psi_{h}=-\int_{0}^{\tau_{b}}\{\frac{1}{\tau_{TOF,h}}+(1-\psi_{0})[\frac{1}{\tau_{Mq,h}}-\frac{\mu_{h}}{\mu_{e}}\frac{1}{\tau_{Mq,e}}]+[\frac{\mu_{h}}{\mu e}\frac{1}{\tau_{Mq,e}}(1-\psi_{0})+\frac{1}{\tau_{Mq,h}}\psi_{0}]\frac{t}{\tau_{b}}\}dt\\ \tau_{b}=\tau_{Mq,h}\frac{\psi_{0}}{\frac{\tau_{Mq,h}}{\tau_{TOF,h}}+\frac{1}{2}} \end{array}$$

Inserting the relation of the differential equation Equation (46) into Equation (51), the current density, obtained for the pure bipolar drift, is expressed as:

(52) $$j=\frac{q}{\tau_{b}}=\frac{q}{\psi_{0}\tau_{Mq,h}}(\frac{\tau_{Mq,h}}{\tau_{TOF,h}}+\frac{1}{2})$$

Thus, the pure bipolar drift leads to an invariable current density, Equation (52), (here charge capture is ignored) with a pulse duration *t_P_* = ψ_0_τ*_Mq,h_*/[(τ*_Mq_*_,*h*_/τ*_TOF_*_,*h*_) + 1/2]. This result indicates that the initial bipolar drift is impossible if the neutral domain is generated directly on the grounded electrode. It is in agreement with the conclusion that the pure monopolar drift of small density electrons is only possible when the generation of carriers is implemented directly at the grounded (low potential) electrode. Pure bipolar drift can be initialized for different injection points ψ_0_ depending on the injected charge amount and ratios of carrier mobilities. These injection points can be predicted by solving of equation:

(53) $$\psi_{0}=\frac{\left(\frac{\mu_{e}}{\mu_{h}}+1)\frac{C_{g}U}{q}+2-(\frac{\mu_{e}}{\mu_{h}}-1\right)}{4(\frac{\mu_{e}}{\mu_{h}}-1)}\left[\sqrt{1+\frac{8(\frac{\mu_{e}}{\mu_{h}}-1)(\frac{C_{g}U}{q}+1)}{{\left((\frac{\mu_{e}}{\mu_{h}}+1)\frac{C_{g}U}{q}+2-(\frac{\mu_{e}}{\mu_{h}}-1)\right)}^{2}}}-1\right]$$

**Figure 3:**
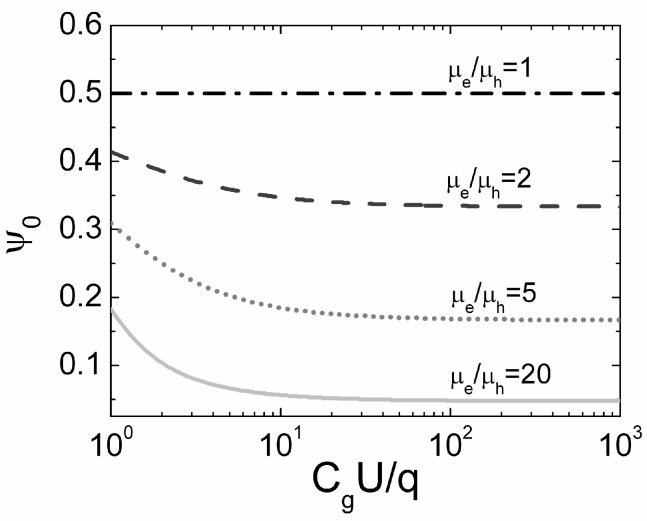
The dependence of the initial position, suitable for the pure bipolar drift, on the ratio of *C_g_U*/*q* simulated for different ratios of electron to hole mobility μ*_e_*/μ*_h_* (μ*_e_*/μ*_h_* = 1 (dash-dotted curve), μ*_e_*/μ*_h_* = 2 (dashed curve), μ*_e_*/μ*_h_* = 5 (dotted curve) and μ*_e_*/μ*_h_* = 20 (solid curve).

The simulated injection point position, for a pure bipolar drift, as a function of *C_g_U*/*q* for different values of mobility ratio μ*_e_*/μ*_h_* is illustrated in Figure 3.

##### Bipolar Domain during Electron Drift Time

In the case of electron drift time, where τ*_tr_*_,*e*_ is the shortest one among characteristic times, a bipolar (τ*_bC_* = τ*_tr_*_,*e*_) drift (Equation (48)) is described by a system of kinetic equations and their solutions, which can be presented as follows:

(54) $$\begin{array}{l} \frac{d\psi_{h}}{dt}=-\frac{\psi_{h}}{\tau_{tr,e}};\;\frac{d\psi_{e}}{dt}=\frac{1-\psi_{0}}{\tau_{tr,e}}\\ \psi_{h}=\psi_{\;0}exp(-\frac{t}{\tau_{tr,e}});\;\psi_{e}=\psi_{0}(1-\frac{t}{\tau_{tr,e}})+\frac{t}{\tau_{tr,e}} \end{array}$$

These solutions satisfy the boundary conditions:

(55) $$\begin{array}{l} \psi_{h}{|}_{t=0}=\psi_{\;0};\;\psi_{e}{|}_{t=0}=\psi_{0}\\ \psi_{h}{|}_{t=\tau_{tr,e}}=\psi_{\;0}exp(-1);\;\psi_{e}{|}_{t=\tau_{tr,e}}=1\\ \;\psi_{h}^{*0}(t=\tau_{tr,e})\equiv \psi_{0}\exp(-1) \end{array}$$

Here, ψ_h_^*^ serves as the start position for hole domain drift, just during the instant of the electron domain disappearing at the high potential electrode. The time τ*_bC_* of the initial bipolar drift is obtained by integrating the expression for drift velocity (using Equations (54) and (55)) as:

(56) $$\frac{d\psi_{e}}{dt}=-\frac{\mu_{e}}{d}E_{2}$$

(57) $$\int_{\psi_{0}}^{1}d\psi_{e}=\int_{0}^{\tau_{tr,e}}\{[\frac{1}{\tau_{TOF,e}}+\frac{\psi_{0}}{\tau_{Mq,e}}]+\frac{\left(1-\psi_{0}\right)}{\tau_{Mq,e}}\frac{\Theta }{\tau_{tr,e}}\}d\Theta -\frac{\mu_{e}}{\mu_{h}}\frac{\psi_{0}}{\tau_{Mq,h}}\int_{0}^{\tau_{tr,e}}\exp(-\frac{\Theta }{\tau_{tr,e}})d\Theta$$

(58) $$\tau_{bC}\equiv \tau_{tr,e}=\frac{(1-\psi_{0})\tau_{Mq,e}}{\frac{\tau_{Mq,e}}{\tau_{TOF,e}}+(\psi_{0}+1)\frac{1}{2}-\psi_{0}[1-\exp(-1)]}\;$$

Current density during bipolar drift is expressed as:

(59) $$j_{bC}(t)=\frac{q}{\tau_{tr.e}}[\psi_{\;0}\;exp(-\frac{t}{\tau_{tr,e}})+(1-\psi_{0})]$$

for the time interval of bipolar drift 0 *≤ t ≤* τ*_tr_*_,*e*_.

For the mixed regime of the bipolar drift during electron monopolar drift, the initial velocity of a large density of holes should be evaluated and charge re-calibration on the electrodes should be performed, to satisfy the conservation of charge and charge momentum (*qv*). The initial velocity of hole drift is obtained using the relations of velocity vectors and their directions for bipolar drift and for the re-calibrated monopolar drift as:

(60) $$\left|{\vec{v}}_{0,h,mon|\psi_{h}^{*0}}\right|=\left|{\vec{v}}_{e,bip,\tau_{b}}\right|+\left|{\vec{v}}_{h,bip,\tau_{b}}{|}_{\psi_{h}^{*0}}\right|=v_{\Sigma bip}$$

This gives a coincidence of *v*_0,*h*,*mon*_ and *v*_Σ*bip*_|_ψh_^*^ values at position ψ_h_^*^ of the hole domain. Thereby, the condition of continuity of the displacement current density is satisfied. It really represents the conservation of charge momentum *qv*.

The induction (⊗) reciprocity is satisfied by *q_e_* on high voltage electrode (*q_e_*⊗1) and *q_h_* on grounded electrode (*q_h_*⊗0) equivalence of the induction on electrodes. The arrival of an electron domain at a high potential electrode (*q_e_*⊗1), requires a re-calibration of charge σ on the high potential electrode, as:

(61) $$\sigma^{+}=\sigma +q_{e}$$

Here, σ^+^ represents a charge on the high potential electrode in the new system coordinates, where the monopolar drift of holes manifests (and can be described by a model presented in Section 2.2.1), just after electrons reach the high potential electrode. To relate velocity in the new system (σ*^+^*) with that in the old one (σ), the re-calibration should be made using Equation (60) for the bipolar drift end-time instant and position ψ_h_^*^ of holes. This happens due to induction of the image charge *q_h_* at ψ = 0 by *q_e_* at ψ = 1 for an instant *t* = τ*_bC_*. The re-calibration of charge on high potential electrode leads to a rearrangement of the field acting on the hole domain during its monopolar drift. Then, the monopolar drift rate is expressed through the old coordinate system parameters. These last re-arranged equations include the charge conservation. The re-calibrations discussed can be generalized as:

(62) $$\begin{array}{l} \sigma^{+}=\sigma +q_{h}\\ \frac{d{\text{ψ}}^{+}}{dt}=\frac{d\text{ψ}}{dt}=\frac{d{\text{ψ}}_{e}}{dt}+\frac{d{\text{ψ}}_{h}}{dt}\\ -{\text{ψ}}^{+}=1-({\text{ψ}}_{h}^{*0}-\text{ψ}) \end{array}$$

Expressions for the monopolar drift time and for a function of ψ (*t*) are obtained by integrating Equation (56) with relevant initial and boundary conditions.

The re-arrangement of the field acting on a hole domain during their monopolar drift is then performed. The monopolar drift rate, expressed through the old coordinate system parameters, is obtained and employed for verification of the coincidence of velocity and current density values at coordinate ψ*_h_* of the old configuration system. There, the signs at scalar quantities are accepted keeping in mind the holes movement direction relative to the system of coordinates (−**k**_0_). The re-calibrations lead to the equivalent coordinate transform relations by making adequate replacements (ψ*_e_^+^* → (1 − ψ*_h_^+^*)). The coordinate transform relations can be obtained by an alternative consideration of prolonged hole drift within re-arranged (**k**_0_ → −**k**_0_^*^, ψ = 0 → ψ^*^ = 1) coordinates.

The generalized expression for current density of the electron bipolar drift prolonged by hole monopolar drift is represented as:

(63) $$j(t)=\{\begin{array}{c} j_{1}=\frac{q}{\tau_{tr,e}}[{\text{ψ}}_{\;0}exp(-\frac{t}{{\text{τ}}_{tr,e}})+(1-{\text{ψ}}_{0})]\text{ for }0\leq t\leq \tau_{tr,e}=\tau_{bC}\;\\ j_{2}=\frac{q_{h}}{{\text{τ}}_{Mq,h}}\exp(\frac{t}{{\text{τ}}_{Mq,h}})[\frac{v_{0,\Sigma bip}}{d}{\text{τ}}_{Mq,h}+\frac{{\text{τ}}_{Mq,h}}{{\text{τ}}_{TOF,h}}-1]\text{ for }0\leq t\leq \tau_{tr,h}=\tau_{tr,h,mon} \end{array}$$

The duration of the entire pulse (*t_P_*) is obtained as a sum of the bipolar drift and hole domain drift, as *t_P_* = τ*_bC_* + τ*_dr_*_,*h*,*mon*_. The current of the bipolar drift as prolonged by the correlated hole drift (the Ramo’s type [8] regime) appears if τ*_TOF_*_,*h*_ = τ*_Mq_*_,*h.*_

In the case of small charge drift, only an approximate description of the process is possible. Then, charge momentum is not conserved for single directional motion. Part of the charge momentum is transferred to charge motion in directions perpendicular to that of the **k**_0_ vector. For small charge drift, *R_h_* = τ*_Mq_*/τ*_TOF_* > 1, the monopolar drift of a hole domain is analyzed in the re-calibrated field using *R_ef_*_,*h*_ instead of *R_h_*. Here, the effective voltage *U_C_*_,*ef*,*h*_ is introduced as:

(64) $$R_{ef,h}=\frac{\tau_{Mq,h}}{\tau_{TOF,h}}\frac{U_{C,ef,h}}{U}=\frac{\tau_{Mq,h}}{\tau_{TOF,h}}\frac{1.06q_{h}d\sqrt[{3}]{\psi_{h}^{*0}}}{\varepsilon \varepsilon_{0}U}$$

for approximation of three-dimensional motion. The coefficient κ*_ef_*_,*h*_ = 1.06 is adjusted for the case μ*_e_* > μ*_h_*. Here, ψ^*^^0^ is the dimensionless location of a drifting domain at the end of a bipolar drift. The initial drift velocity of the monopolar drift is equal to that acquired by a sub-domain instantaneous velocity at ψ^*^^0^ and at the end (τ*_dr_*_,*b*_) of the bipolar drift. The applicability of the *U_C_*_,*ef*,*e*_ approximation models has been verified by their relevance in bringing together the one-dimensional solutions of bipolar and monopolar drift, thereby closer matching the synchronous conservation of charge, charge momentum and current density continuity. For regimes close to those of Ramo’s, *i.e.*, for 0.9 < *q*/*C_g_U* ≤ 1, it can be always kept *R_h_* = 1. Alternatively to the *R_ef_*_,*h*_ and *U_C_*_,*ef*,*e*_ approach, the retardation and magnetic field effects can be analyzed [10,23,24,25,26,27].

Similar consideration of the mixed drift regime with the faster arrival of the hole domain to the grounded electrode can be performed. The real evolution (the rise to peak) of the current density should be considered by including the process of capacitance charging. Current density decreases after the initial peak during the bipolar drift of separate domains due to drag of a late arrival sub-domain caused by induction. However, it only contains the increasing component after, for instance holes are extracted at the grounded electrode, and only the monopolar drift of the electron domain then exists.

In the case of small charge drift and primary arrival of electron domain, only an approximate description of the process is possible. Then, charge momentum is not conserved for motion in a single direction. Part of the charge momentum is transferred to charge motion in directions perpendicular to that of the **k**_0_ vector. Therefore, a convergence (stitch) of velocity values at the instant of the change of drift regime from the bipolar to a monopolar one, should be performed. Synchronously, the deviation of the ratio of characteristic times *R_e_*(ψ, *U*) = τ*_Mq_*_,*e*_/τ*_TOF_*_,*e*_ from unity (from the correlated drift, the Ramo’s type regime), dependent on drifting charge amount and voltage sharing (on the instantaneous domain position) should be evaluated.

For mixed regime of the small charge drift (*R_e_* = τ*_Mq_*/τ*_TOF_* > 1) and primary arrival of electron sub-domain to high potential electrode, the charge re-calibration and initial drift velocity evaluation by *v*_0_*|*_ψ_^*0^ = *|v_eb_*| + |*v_hb_|* procedures are performed. Then, the monopolar drift is analyzed in the re-calibrated field, using *R_ef_*_,*e*_ instead of *R_e_*. Here, the effective voltage *U_C_*_,*ef*,*e*_, which governs the monopolar drift of electrons, is introduced into *R_ef_*_,*e*_ expression by:

(65) $$R_{ef,e}=\frac{\tau_{Mq,e}}{\tau_{TOF,e}}\frac{U_{C,ef,e}}{U}=\frac{\tau_{Mq,e}}{\tau_{TOF,e}}\frac{1.13q_{e}d\sqrt[{3}]{\psi_{e}^{*0}}}{\varepsilon \varepsilon_{0}U}$$

for approximation of the three-dimensional motion. The initial drift velocity of the monopolar drift is equal to that acquired by a sub-domain instantaneous velocity at ψ_e_^*^^0^ and at the end (τ*_tr h_*) of the bipolar drift. The applicability of the *U_C_*_,*ef*,*e*_ approximation models has again been verified by their relevance in bringing together the one-dimensional solutions of bipolar and monopolar drift, thereby closer matching the synchronous conservation of charge, charge momentum and current density continuity.

### 2.3. Induced Charge Drift Current Transient in Junction Type Detectors

The slightly different consideration of partially and fully depleted junction structures is performed. More detail description of these situations is presented in our publications [17,20]. Here, the essential features of operation dynamics in junction type detectors are briefly discussed for analysis of the impact of carrier scattering and multiplication effects.

#### 2.3.1. Injected Charge Drift Current Transients in Partially Depleted Detector Base

The analysis is performed by consideration of the barrier capacitance changes for unit area *C_b_* = εε_0_/*w_q_*(*t*) (equivalent to *C_Sq_*). The charge drift dependent depletion width *w_q_*(*t*) then appears due to the injected charge *q_e_*. The reverse biased steady-state depletion width *w*_0_ = [2εε_0_(*U* + *U_bi_*)/*eN_Def_*]^1/2^ serves as an equivalent of the inter-electrode spacing *d*. Variations of surface charge σ are equivalent to the changes of the surface charge at *w*_0_, induced by an additional depletion charge bar, as σ ~ *eN_Def_*(*w_q_*(*t*) − *w*_0_). Thus the time dependent changes of the system dynamic capacitance *C_Sq_* (ascribed to a surface area unit) is equivalent to the analysis of the convection current.

Detailed derivation of the main relations and discussion of regimes is presented in [17,28]. For the range of the applied reverse voltages *U_bi_* < *U* < *U_FD_* on the n-type conductivity base and using an assumption that the electron domain is injected nearby the metallurgic abrupt junction, the electric field is capable to separate electron-hole pairs and to extract holes into p^+^-type emitter. This leads to a synchronous change of the depletion widths in the n- and p- type conductivity layers to keep the junction system electrically neutral behind the depletion *w*_0,*n*_ and *w_p+_* width boundaries. To simplify the analysis, an assumption of the asymmetric doping of n- and p-layers, *i.e.*, the abrupt p-i-n junction is accepted, which enables ones to ignore a voltage drop on p^+^-layer emitter.

Using the methodology described in [17,28], an instantaneous field distribution and depletion width changes dependent on the instantaneous position of the injected domain are obtained by taking Poisson integrals. An instantaneous depletion width *w_q_* and, alternatively, *E*_1_(0), can be expressed as:

(66) $$w_{q}=\frac{\varepsilon \varepsilon_{0}}{eN_{Def}}(E_{1}(0)+\frac{q_{e}}{\varepsilon \varepsilon_{0}})=\sqrt{\frac{2\varepsilon \varepsilon_{0}}{eN_{Def}}[U+\frac{q_{e}}{\varepsilon \varepsilon_{0}}X_{e}]\;}=w_{0}\sqrt{\left(1+\frac{w_{0}^{2}\mu_{e}q_{e}/w_{0}}{\mu_{e}U\varepsilon \varepsilon_{0}}\frac{X_{e}}{w_{0}}\right)}=w_{0}\sqrt{1+\frac{\tau_{TOF,w_{0}}}{\tau_{Mq,w_{0}}}\frac{X_{e}}{w_{0}}}$$

and:

(67) $$E_{1}(0)=\frac{eN_{Def}}{{\text{εε}}_{0}}\sqrt{\frac{2{\text{εε}}_{0}}{eN_{Def}}[U+\frac{q_{e}}{{\text{εε}}_{0}}X_{e}]}-\frac{q_{e}}{{\text{εε}}_{0}}$$

Here, a common depletion boundary condition (*E*(*w_q_*) = 0) and a proper root of the quadratic equation are accepted; μ denotes the carrier mobility. For the reverse biased junction, it is commonly [29] kept that *U* = *U_r_* − *U_bi_*. Within a still system of coordinates, the characteristic time parameters τ*_Mq_*_,*w*0_ and τ*_TOF_*_,*w*0_, are defined relative to a steady-state width *w*_0_, instead of *d*. Thereby, the characteristic time parameters τ*_Mq_*_,*w*0_ and τ*_TOF_*_,*w*0_, are expressed as:

(68) $$\tau_{TOF,w0}=\frac{w_{0}^{2}}{\mu_{e}U}$$

(69) $$\tau_{Mq,w0}=\frac{\varepsilon \varepsilon_{0}}{\mu_{e}(q_{e}/w_{0})}$$

These characteristic times, namely, their equality (τ*_Mq_*_,*w*0_ = τ*_TOF_*_,*w*0_), can be a measure for the validity of the electrostatic induction approach. A steady-state depletion width *w*_0_ is expressed by a well-known formula derived within depletion approximation [17,29] as *w*_0_ = (2εε_0_*U*/*eN_D_*)^1/2^. Consequently, a barrier capacitance is obtained as:

(70) $$C_{b,Sq}=\frac{{\text{εε}}_{0}}{w_{q}}\equiv \frac{{\text{εε}}_{0}}{w_{0}\sqrt{1+\frac{{\text{τ}}_{TOF,w_{0}}}{{\text{τ}}_{Mq,w_{0}}}\frac{X_{e}}{w_{0}}}}=\frac{C_{b0}}{\sqrt{1+\frac{{\text{τ}}_{TOF,w_{0}}}{{\text{τ}}_{Mq,w_{0}}}\frac{X_{e}}{w_{0}}}}$$

where *C_b_*_0_ = εε_0_/*w*_0_. It can be noticed that *w_q_* > *w*_0_, and, therefore, the barrier capacitance decreases due to the injected charge domain. The expression of a module of the current density of the injected charge domain (ICD) drift can then be represented as follows:

(71) $$\left|i_{ICD}\right|=q_{e}\frac{S}{w_{0}}\frac{1}{2{\left(1+\frac{{\text{τ}}_{TOF,w_{0}}}{{\text{τ}}_{Mq,w_{0}}}\frac{X_{e}}{w_{0}}\right)}^{3/2}}\frac{dX_{e}}{dt}=q_{e}\frac{S}{w_{0}}K_{\tau }\frac{dX_{e}}{dt}=q_{e}K_{\tau }S\frac{d{\text{ψ}}_{w_{0}}}{dt}$$

The obtained scalar form of the current within a still system of coordinates (*w*_0_) is very similar to that of the Ramo’s current [8] expression. The main difference is an appearance of a coefficient *K*_τ_, dependent on the dimensionless position ψ^*^ = *X_e_*/*w*_0_ of a drifting surface charge domain within *w*_0_, and it is composed of the characteristic times as:

(72) $$K_{\tau }=\frac{1}{2{\left(1+\frac{{\text{τ}}_{TOF,w_{0}}}{{\text{τ}}_{Mq,w_{0}}}{\text{ψ}}^{*}\right)}^{3/2}}$$

The coefficient *K*_τ_ determines a specific feature of the non-fixed position of the virtual electrode (*w_q_*), charge on which is varied by a changed depletion range of ions. The possible drift length is also dependent on the rate of the formation of *w*_0_ and *w_q_*, *i.e.*, on the characteristic time τ*_Nd_*_,*ef*_ = εε_0_/*e*μ*N_D_*_,*ef*_ = εε_0_/*e*μ*n_ENR_* of the stabilization of the transitional λ-thick layer (between the depletion and ENR layers) due to extraction of the mobile carriers *n_ENR_* = *N_D_*_,*ef*_ from ENR.

The additional scalar equation for the instantaneous velocity of the charge domain drift is expressed (within the dimensionless ψ^*^ = *X_e_*/*w*_0_ form) as:

(73) $$\frac{d\psi^{*}}{dt}=\frac{1}{\tau_{M,Ndef}}[\sqrt{1+\frac{\tau_{TOF,w_{0}}}{\tau_{Mq,w_{0}}}\psi^{*}}-\psi^{*}]$$

with the adequate boundary conditions, as *t* = 0 for ψ^*^ = ψ_0_^*^ and *t* = *t_dr_* for ψ^*^ = 1, respectively. The solution of Equation (73):

(74) $$t=\tau_{M,Ndef}[\int_{\psi_{0}^{*}}^{\psi^{*}(t)}\frac{1}{\sqrt{1+\xi \frac{\tau_{TOF,w_{0}}}{\tau_{Mq,w_{0}}}}-\xi }d\xi ]$$

can be found numerically [22]. Evaluation of drift time *t_dr_*, is implemented by inserting the second boundary condition ψ^*^ = 1:

(75) $$t_{dr}={\text{τ}}_{M,Ndef}[\int_{\psi_{0}^{*}}^{1}\frac{1}{\sqrt{1+\text{ξ}\frac{{\text{τ}}_{TOF,w_{0}}}{{\text{τ}}_{Mq,w_{0}}}}-\text{ξ}}d\text{ξ}]$$

Thereby, time dependent variation of an initial component of current *i_ICD_*_,*F*_(*t*) is described for the time interval 0 ≤ *t* ≤ *t_dr_* as:

(76) $$i_{ICD}{,}_{F}(t)=\frac{q_{e}S}{2{\text{τ}}_{M,Ndef}}\frac{\left[\sqrt{1+\frac{{\text{τ}}_{TOF,w_{0}}}{{\text{τ}}_{Mq,w_{0}}}{\text{ψ}}^{*}(t)}-{\text{ψ}}^{*}(t)\right]}{{\left[1+\frac{{\text{τ}}_{TOF,w_{0}}}{{\text{τ}}_{Mq,w_{0}}}{\text{ψ}}^{*}(t)\right]}^{3/2}}$$

**Figure 4:**
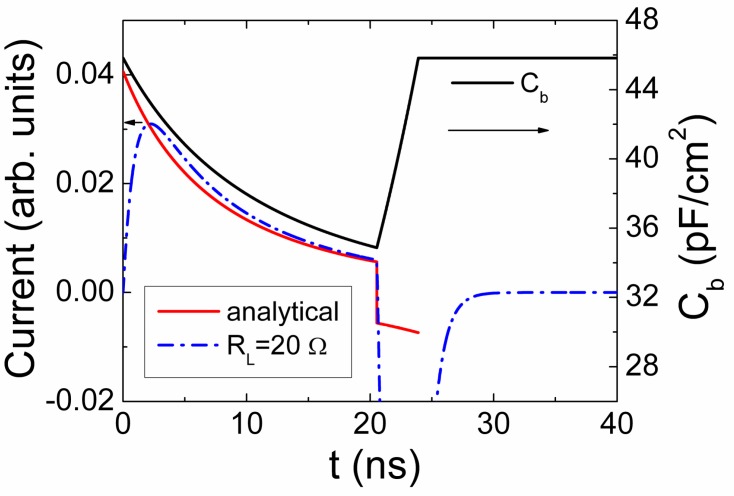
Current density transients simulated without including of external circuit impact (red solid curve) and under the impact of external circuit with *R_L_* = 20 Ω (dash-dotted blue curve). Solid black curve represents the barrier capacitance variations during charge drift.

This expression describes a pulse with a decreasing current value within a pulse vertex, Figure 4.

The rearward component (Figure 4) of the current pulse is determined by the processes of the system capacitance *C_b_*_,*Sq*_ restore to its steady-state value *C_b_*_,*S*0_ = εε_0_/*w*_0_ [18]. This process originates a current increase within a rearward phase of current pulse, and it appears due to narrowing of the depletion region. The mentioned processes lead to the differentiation effect (Figure 4) when voltage on partially depleted device is varied due to voltage drop on load resistor. The estimation of barrier capacitance and depletion width of the partially depleted junction can be performed using analysis of the current pulse shape, amplitude and duration (Figure 4).

#### 2.3.2. Induced Charge Drift Current Transients in Fully Depleted Detector

The surface charge on a metallic/heavily doped electrode changes together with the space charge bar width due to a moving surface charge domain, if the external voltage *U* is equal to or exceeds the full depletion (*U_FD_*) value, *U* ≥ *U_FD_*. This happens due to a lack of mobile carriers to withstand an action of the injected charge determined electric field. Thereby, the equilibrium carriers are extracted into the external electrode, if depletion covers completely the active layer width. The detail derivations of the expressions for acting fields, surface charges and current density are published in [17].

A module of the current, in the case of the over full-depleted (OFD) junction, is expressed as:

(77) $$i_{OFD}(t)=S\frac{d\text{σ}}{dt}=q_{e}\frac{S}{d}\frac{dX_{e}}{dt}$$

which formally represents the Ramo’s type [8] current component. The complete current within an external circuit is also determined by the displacement current due to change of drifting charge instantaneous position. This displacement current exactly compensates the conductivity (convection) current component in a fully depleted junction layer.

A dimensionless velocity, determined as instantaneous position changes normalized to a geometric width *d* of the inter-electrode spacing, is expressed as:

(78) $$\frac{v(X_{e})}{d}=\frac{d\psi }{dt}=\mu_{e}[\frac{U}{d^{2}}+\frac{1}{2}\frac{eN_{Def}}{\varepsilon \varepsilon_{0}}-\frac{eN_{Def}}{\varepsilon \varepsilon_{0}}\psi +\frac{q_{e}}{\varepsilon \varepsilon_{0}d}\psi ]=\frac{1}{\tau_{TOF}}+\frac{1}{2\tau_{M,NDef}}+\psi \left(\frac{1}{\tau_{Mq}}-\frac{1}{\tau_{M,NDef}}\right)$$

The coefficients in equation Equation (78) can easily be rearranged by using the characteristic time parameters:

(79) $$\begin{array}{l} {\text{τ}}_{TOF}=\frac{d^{2}}{{\text{μ}}_{e}U}\\ {\text{τ}}_{M,NDef}=\frac{{\text{εε}}_{0}}{{\text{μ}}_{e}eN_{Def}}\\ {\text{τ}}_{Mq}=\frac{{\text{εε}}_{0}}{{\text{μ}}_{e}(q_{e}/d)} \end{array}$$

and their ratios. As shown in [17], three type solutions and regimes are then obtained.

The current ascribed to different regimes can be modelled by using the relevant expressions for ψ(*t*), taken from the drift velocity equation (Equation (78)). The current for different regimes [17] is expressed as:

(80A) $$i_{OFD}(t)=\frac{q_{e}S}{{\text{τ}}_{Mq}}[(\frac{{\text{τ}}_{Mq}}{{\text{τ}}_{TOF}}+\frac{{\text{τ}}_{Mq}}{2{\text{τ}}_{M,Ndef}})+(1-\frac{{\text{τ}}_{Mq}}{{\text{τ}}_{M,Ndef}})\{{\text{ψ}}_{0}\exp[-\frac{t}{{\text{τ}}_{Mq}}(\frac{{\text{τ}}_{Mq}}{{\text{τ}}_{M,N_{Def}}}-1)]+\frac{\frac{1}{{\text{τ}}_{TOF}}+\frac{1}{2{\text{τ}}_{M,Ndef}}}{\left(\frac{1}{{\text{τ}}_{M,N_{Def}}}-\frac{1}{{\text{τ}}_{Mq}}\right)}\{1-\exp[-\frac{t}{{\text{τ}}_{Mq}}(\frac{{\text{τ}}_{Mq}}{{\text{τ}}_{M,N_{Def}}}-1)]\}\}],\text{ for }{\text{ τ}}_{Mq}>{\text{τ}}_{M,N_{Def}}$$

(80B) $$i_{OFD}(t)=q_{e}S(\frac{1}{{\text{τ}}_{TOF}}+\frac{1}{2{\text{τ}}_{M,N_{Def}}}),\text{ for }{\text{τ}}_{Mq}={\text{τ}}_{M,N_{Def}}$$

(80C) $$i_{OFD}(t)=\frac{q_{e}S}{{\text{τ}}_{Mq}}[(\frac{{\text{τ}}_{Mq}}{{\text{τ}}_{TOF}}+\frac{{\text{τ}}_{Mq}}{2{\text{τ}}_{M,Ndef}})+(1-\frac{{\text{τ}}_{Mq}}{{\text{τ}}_{M,Ndef}})\{{\text{ψ}}_{0}\exp[\frac{t}{{\text{τ}}_{Mq}}(1-\frac{{\text{τ}}_{Mq}}{{\text{τ}}_{M,N_{Def}}})]+\frac{\frac{1}{{\text{τ}}_{TOF}}+\frac{1}{2{\text{τ}}_{M,Ndef}}}{\left(\frac{1}{{\text{τ}}_{Mq}}-\frac{1}{{\text{τ}}_{M,N_{Def}}}\right)}\{\exp[\frac{t}{{\text{τ}}_{Mq}}(1-\frac{{\text{τ}}_{Mq}}{{\text{τ}}_{M,N_{Def}}})]-1\}\}],\text{ for }{\text{τ}}_{TOF}≅{\text{ τ}}_{Mq}<{\text{τ}}_{M,N_{Def}}$$

The current changes within a pulse vertex acquire a relaxation curve shape for the regime A, when screening of electrons by ion charge within the depletion width prevails. For the correlated screening regime B, a square-wave shape current pulse appears with a flat vertex. While for the correlated (Ramo’s type) drift regime C (τ*_TOF_* = τ*_Mq_*), the transient with increasing in time current is inherent. The monopolar drift of holes can be expressed using methodology described above for the case of electrons drift in capacitor type detector.

Impact of ion space charge in fully depleted detector can be deduced from analysis of Equations (79) and (80). The moving charge inside the over depleted space charge layer induces a displacement current component, which exactly compensates the conductivity current component, arisen due to a proximate contacting of the depleted layer with external electrode (outside layer). As can be inferred for the regime B (Equation (80B)), characterized by the matched relaxation lifetimes τ*_Mq_ =* τ*_M_*_,*NDef*_, the space charge *eN_Def_* over *d* accelerates a drift of the injected charge domain by the shortening of the drift time to the value *t_dr_* = τ*_TOF_*/[1 + (τ_*TOF*_/2τ_*M,Ndef*_)] < τ*_TOF_*, for ψ_0_ = 0 (Equation (80B)). The large injected charge is able to locally screen the space charge of ions, for the regime C (Equation (80C)). Then, a drift of the injected domain proceeds similarly to that in a capacitor-type device.

Analysis of the bipolar drift of a surface charge in junction structure is performed by using the expressions for acting fields, surface charges and current density, published in [17,20]. The induced charge current density, due to a bipolar drift, is expressed as follows:

(81) $$i=S\frac{d\text{σ}}{dt}=-(q_{h}\frac{1}{d}\frac{dX_{h}}{dt}-q_{e}\frac{1}{d}\frac{dX_{e}}{dt})S\;$$

It can be noticed that, owing to **v**_e_ = −**v**_h_, the scalar current can be represented by a sum of Ramo’s-type [8] components:

(82) $$i=q(\frac{d\psi_{h}}{dt}+\frac{d\psi_{e}}{dt})S$$

The pure bipolar and mixed drift regimes are considered [17] similarly to those discussed for capacitor type detectors.

## 3. Large and Small Charge Drift Regimes

In our consideration, the main equation for the current density (for instance, considering the motion of the electron monopolar domain) can be directly obtained by using the electrostatic energy balance:

(83) $$\text{δ}(\text{σ}U)=-\text{δ}(q_{e}\Phi )$$

Here, δ means a change in the electrostatic energy due to a variation of the surface charge (δσ) on the electrode, which should be balanced by a change in the energy of the moving charge *q_e_*. The latter *q_e_* is assumed to be invariable, and these energy changes are ascribed to the surrounding equal-potential δΦ(*X*_e_) changes (the Φ surface shape must be invariable, *i.e.*, not deformed, within Ramo’s [8] approach) during charge drift. The temporal changes of the surface charge on the electrode gives current density variations dependent on time (for the fixed external voltage), and this current is generally expressed as:

(84) $$i(t)=S\frac{d\text{σ}}{dt}=-q_{e}\frac{S}{U}\frac{\partial \Phi }{\partial X_{e}}\frac{\partial X_{e}}{\partial t}$$

This Equation (84) coincides entirely with Ramo’s current derivation. Accepting the general electrostatic relation **E** = −gradΦ *=* **n**_e_*q_e_/*εε_0_ = **E**_q_ (for the instantaneous surface charge field vector) and assuming that *E_q_* = *q_e_*/εε_0_, for its scalar representation, the Equation (16) is rearranged as:

(85) $$i(t)=S\frac{d\text{σ}}{dt}=q_{e}\frac{S}{U}\frac{q_{e}}{{\text{εε}}_{0}}\frac{\partial X_{e}}{\partial t}=q_{e}\frac{d^{2}}{U}\frac{S}{d}\frac{q_{e}}{{\text{εε}}_{0}}\frac{\partial X_{e}/d}{\partial t}=q_{e}\frac{d^{2}}{U}\frac{S}{d}\frac{q_{e}}{{\text{εε}}_{0}}\frac{d{\text{ψ}}_{e}}{dt}$$

Let’s denote the scalar values of the field within the capacitor inter-electrode space as *E*_σ_ = *U*/*d* and div**E**_q_ = (**∇ n**_e_*q_e_*ψ_e_/εε_0_) = *q_e_*/εε_0_*d*, for the moving injected charge field *E_q_* (over a geometrical width *d*). Assuming a balance of the instantaneous electric fields *E*_σ_ and *E_q_* (*E*_σ_ = *E_q_*) a weighing field *W_E_* can be formally introduced as *W_E_* = div**E**_q_*/E*_σ_ = *d*^−1^. Introduction of a weighting field for the considered plane-parallel symmetry system is rather artificial. This, perhaps, has some sense for the Ramo’s analyzed system. There, a spherically- symmetrical equi-potential (surrounding the elementary charge) changes (*d*Φ/*dX_e_*) due to charge drift and the crossing of the infinite electrode surface plane. Therefore, a weighting field is needed to balance the geometrical measures. In our case, projection of the radius-vector **r** (in Φ(*r*)) and of the surface normal **n**_e_ onto the charge motion axis **k**_0_ coincides with these **r** and **n**_e_ vectors. Therefore, the parameter of a dimensionless position ψ*_e_* = *X_e_*/*d* is more reasonable, without the introduction of the artificial weighting field. However, the infinitesimal width of the surface charge domain should be assumed, which has no parallel (to motion direction) boundary. The assumptions discussed lead to an equality of fields at any instant and *q_e_* location, as *E*_σ_ = *U*/*d* = *E_q_* = *q_e_*/εε_0_. This relation is valid, as mentioned, for a small external voltage (as assumed in Ramo’s derivation) or a drifting charge domain of large density, to completely terminate the field of the local charge on the electrode by the drifting one. The result obtained can be explained by the equal action and re-action of the surface (σ and *q*) charges. The electrostatic approach (τ*_TOF_ =* τ*_Mq_*) is based on the superposition of the acting fields *E*_σ_ and *E_q_*, without any interaction between them (*E*_σ_∙*E_q_* = 0), as the material is assumed to be linear. The latter assumption excludes any mediation for the *E*_σ_∙*E_q_* interplay. Then, Equation (85) can be further rearranged (at assumption *E*_σ_ *= E_q_*, equivalent to the equality of response times τ*_Mq_*_,*e*_ = τ*_TOF_*_,*e*_ within the precision of the electrostatic approach) as:

(86) $$i(t)=S\frac{d\text{σ}}{dt}=q_{e}\frac{d^{2}}{{\text{μ}}_{e}U}\frac{S}{d}\frac{{\text{μ}}_{e}q_{e}}{{\text{εε}}_{0}}\frac{d{\text{ψ}}_{e}}{dt}=q_{e}S\frac{{\text{τ}}_{TOF,e}}{{\text{τ}}_{Mq,e}}\frac{d{\text{ψ}}_{e}}{dt}=q_{e}S\frac{d\text{ψ}(X_{e})}{dt}$$

Unfortunately, consideration of the energy balance gives no recipes for determining the drift velocity field *v_dr_*(*X*_e_) = *dX*_e_/*dt*. To find the distribution of the drift velocity field, the problem should be solved by consideration of the fields and charges in detail and by analyzing the different charge injection and drift situations.

The scalar expressions for a surface charge can be re-arranged, using the characteristic times τ*_TOF_* and τ*_Mq_*, presented in Equations (79). The introduced drifting charge dielectric relaxation time τ*_Mq_*_,*e*,*h*_ determines a reaction time (during which charge *q* is able to change its position to balance the action of the electrode charge) of the drifting charge in the volume (*Sd*) surrounded by the electrodes. This τ*_Mq_*_,*e*,*h*_ reciprocally depends on the induced surface charge *q_e_*_,*h*_ density. Introduction of the characteristic times is more convenient and essential for analysis of drift velocity field in the dimensionless coordinate system.

The free flight time τ*_TOF_* (under the action of the Coulomb force created by the surface charge on the electrode) characterizes the ability (rate) of the electrode’s field to provide or change the velocity of the drifting charge *q* over a specific length *r* = *d* (*i.e.*, ψ = 1). This ability depends on the amount of drifting charge. The τ*_TOF_* representation by Equations (79) is only valid for the correlated drift (Ramo’s [8] regime). The diffusion processes, retardation and magnetic field effects should be included if the injected charge can not be completely and rapidly enough terminated by the charge on the electrode. This can happen at the boundaries of plates of a capacitor. The diffusion processes may be either caused by a corrugated surface or by the sharp lateral gradients inherent for the small surface charge domains. In real structures of finite dimensions *d_x_*, *d_y_*, *d_z_*, a discrete spectrum of diffusion (with carrier diffusion coefficient *D*) governed carrier density decay modes is inherent [30,31,32,33], and it is characterized by the respective spatial frequencies of η,ξ, ζ*.* Spatial frequencies η, ξ, ζ appear in the solutions of the two-side boundary problem for the task of the diffusion (discussed later) and can be understood by the analogy of the sinη*x* with frequency in time domain as sinω*t*. This leads to sufficiently rapid (in the time scale of τ_η,ξ,ζ_ = 1/η*_m_*^2^*D*, 1/ξ*_m_*^2^*D*, 1/ζ*_m_*^2^*D*) carrier movements with their instantaneous velocities *v* ~ η*D*, ξ*D*, ζ*D*, especially for the initial drift instants, when τ_η,ξ,ζ_ → 0, due to *m* → ∞. The diffusion processes can also prevail if the injected charge is larger than the charge created on the electrode by the battery. Alternatively, diffusion is important if the velocity (*v_x_*_,*y*,*z*_ ~ η*D*, ξ*D*, ζ*D*) of the carrier movement under the gradients is larger than that provided by the external voltage in the range of small distances (large spatial frequencies η, ξ, ζ). Therefore, the rate 1/τ*_TOF_* of changes, governed by electrostatic field, should be evaluated by either considering the dynamic or kinetic equation of motion of small charge as well as the mechanical/electrostatic energy transformation/conservation conditions. Then, the characteristic time τ*_TOF_* depends on acting voltage *U*^−α^ with α in the range of α = ½ − 1, and it can be a function of time τ*_TOF_* = *f*(*t*) for different approximations of the experimental/detection situations. Therefore, the simple definition of τ*_TOF_* (Equations (79)) is insufficient to completely characterize the drift kinetic in general, due to necessity of the correlated drift. As shown in Section 2.2, neither the domain velocity nor acceleration is constant in the general case, if ψ(*t*) *~* exp(*t*/τ*_Mq_*). The more complicated drift of a small charge is obtained when the three-dimensional motion of carriers is included. Then, additional specific time parameters should be introduced to characterize carrier diffusion instantaneous lifetimes τ*_Dy_* = 1/ξ*_m_*^2^*D* or τ*_Dz_* = 1/ζ*_w_*^2^*D*, and velocities *v_y_* = ξ*_m_D* or *v_z_* = ζ*_w_D*.

**Figure 5:**
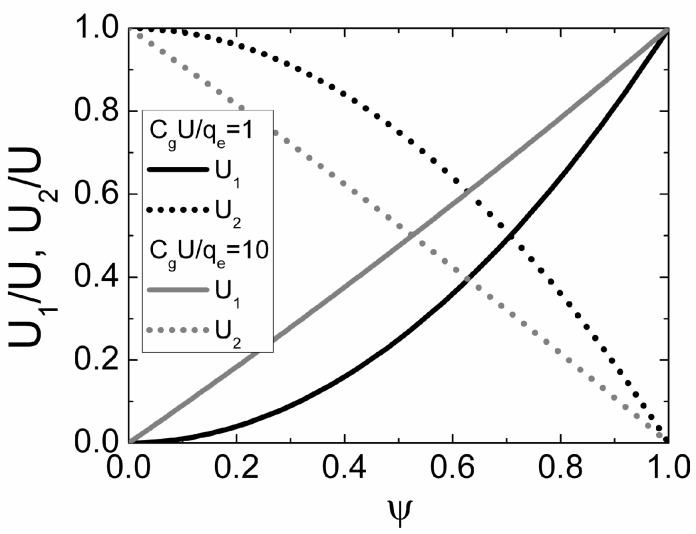
Normalized voltage *U*_1_/*U* (solid curves) and *U*_2_/*U* (dotted curves) drop within the different regions of an inter-electrode gap during the drift of charge *q* for *C_g_U*/*q* = 1 (black curves) and *C_g_U*/*q* = 10 (grey curves) for electrons.

Nevertheless, by employing the characteristic time parameters τ*_Mq_*_,*e*_ and τ*_TOF_*_,*e*_ and effective ratio coefficients *R_ef,e_*, the drift determined current density can be simply expressed by Equation (86). It can be inferred that Ramo’s regime appears if τ*_Mq_*_,*e*_/τ*_TOF_*_,*e*_ = 1. Actually, this means a large drifting charge *q_e_ =* σ|_t=0_ = *C_g_U = q_C_.* This drifting charge *q_e_* is able to terminate the action of electrode charge σ at any instant and location of *q_e_*. Only the equality of the action and reaction times (reciprocity in time) ensures the conservation of energy. The Ramo’s regime for a finite area (*S*) of electrodes is equivalent to the large charge (in comparison with *q_C_* = *C_g_U* = *q_e_*_,*h*_) drift. This implies that the electrostatic field, induced by a surface charge σ = *q_C_* and created by an external voltage *U*, is completely terminated on the drifting charge domain *q_e_*. Therefore, all the applied voltage *U* drops within the gap between the high potential electrode and the *q_e_* domain. The drift of a large monopolar charge domain is only possible if the positive charge *q_C_* (on the high potential electrode) is completely (and over all the drift instants (τ*_TOF_*_,*e*_/τ*_Mq_*_,*e*_ = 1)) balanced by the electro-statically induced positive charge −*q_e_* → *+*|*q_e_*|. (The injected charge –*q_e_* field direction in Figure 1 can be alternatively understood as a field ascribed to the induction charge +|*q_e_*|). This leads to the necessity of the complete electrostatic energy balance. Due to domain (*q_e_*) drift, the gap between the high potential electrode and the domain decreases from ψ = (1 − ψ_0_) to ψ = 0. Therefore, a drifting domain additionally acts as a voltage sharing element with the parabolic-like characteristic of *U_2_* = (1 *−* ψ^2^)*U*, Figure 5. For the small charge domain, the acting voltage *U_C_* is less than *U*, and a voltage divider exhibits the linear characteristic, Figure 5.

In real detectors, the prevailing regime is the detection and collection of a small drifting charge, when τ*_Mq_*_,*e*_/τ*_TOF_*_,*e*_ > 1. Unfortunately, this regime can only be approximately considered within the one-dimensional approach. The reason is a slow dielectric relaxation (τ*_Mq_*_,*e*_/τ*_TOF_*_,*e*_ > 1) of a small drifting charge *q_e_*. Due to the small *q_e_*, the charge domain surface becomes corrugated under the action of the electrode charge and the charge density gradients within the domain plane. To stabilize the gradients (or the oblique action of σ surface boundary segments), the drifting charge should vary its position in all three spatial dimensions. Thus, the lateral fields should be taken into account. On the basis of the Lagrange variational principle, it can be understood that charge movements within both the electrode and domain planes should be correlated, to react most rapidly to each others’ changes. Then, energy conservation can only be considered by an analysis of the three dimensional charge drift and diffusion problem. This leads to the appearance of charges and their neutralization currents on the perpendicular (to an inter-electrode drift direction) boundary planes of the inter-electrode dielectric.

Nevertheless, a few estimations could even be made on the basis of the energy balance consideration in one dimensional approach. For the Ramo’s regime of the correlated drift, *i.e.*, for instantaneous relation *U*/*d* = *q_e_*/εε_0_, and a large density of *q_e_*, the current density within the pulsed *I-V* characteristic can be represented as:

(87A) $$j(U)=q_{e}\frac{d}{U}\frac{1}{d}\frac{q_{e}}{{\text{εε}}_{0}}\frac{\partial X_{e}}{\partial t}=q_{e}\frac{d}{U}\frac{1}{d}\frac{q_{e}}{{\text{εε}}_{0}}{\text{μ}}_{e}\frac{U}{d}={\text{εε}}_{0}{\text{μ}}_{e}\frac{U^{2}}{d^{3}}\text{ when }v_{dr}{|}_{X_{e}=0}={\text{μ}}_{e}\frac{U}{d}$$

(87B) $$j(U)=\frac{9}{8}{\text{εε}}_{0}{\text{μ}}_{e}\frac{U^{2}}{d^{3}}\text{ when }v_{dr{|}_{X_{e}=\frac{1}{2}d}}={\text{μ}}_{e}(\frac{U}{d}+\frac{q_{e}}{2{\text{εε}}_{0}})=\frac{3}{2}{\text{μ}}_{e}\frac{U}{d}$$

(87C) $$j(U)=4{\text{εε}}_{0}\mu_{e}\frac{U^{2}}{d^{3}}\text{ when }v_{dr{|}_{X_{e}=d}}={\text{μ}}_{e}(\frac{U}{d}+\frac{q_{e}}{{\text{εε}}_{0}})=2{\text{μ}}_{e}\frac{U}{d}$$

It can be noticed in Equations (87), that for the regime of a large charge drift, the current density is a parabolic function of the voltage. This is in agreement with the voltage sharing effect (Figure 5). This means that equation Equation (87B) can be associated with an average of the drift range (*i.e.*, ψ = 1/2) among values assumed in Equations (87B) and (87C), respectively. This expression (Equation (87B)) coincides with that for the space charge limited current derived by Mott-Gurney [34,35,36] for dielectrics.

The combined analysis of the electrostatic energy balance discussed above, and the direct solution of the field and kinetic equations enable ones to make estimations of the drift current dependence on the applied voltage. There dependences on the injected charge amount and its drift velocity are also described. The obtained solutions exactly coincide with those derived from Ramo’s theorem [8,9] and (Equations (87)) Mott-Gurney’s law [35,36]. Thereby, the correlated drift (Ramo’s regime) is defined by the assumptions and consequences of Shockley- Ramo’s theorem [8,9]: the electrostatic approach τ*_TOF_* = τ*_Mq_* is valid; the boundary effects (electric field distortion) can be ignored; the charge, charge momentum and electrostatic energy conservation is valid; the expression of the convection (drift) current obeys the Ramo’s law, as *j* = *q_e_*dψ*_e_*/d*t,* and the Mott-Gurney’s law [35,36] *j ~ U*^2^/*d*^3^.

The dynamic capacitance of a system *C_S_*_,*q*_ depends on the instantaneous location ψ*_e_* of the surface charge domain within the inter-electrode space, and it changes during the motion of this domain. However, it depends on the system relaxation characteristic times (from another point of view, on the induced charge and the applied voltage) to keep the electrostatic fields and energy balanced. The capacitance of the system initially decreases due to the injection of a drifting charge *q_e_*, relative to its value without the induced charge. This is caused by the reduction of the surface charge on the electrode σ which is involved in the termination of the moving surface charge *q_e_*. However, the requirement for non-negative *C_S_*_,*q*_ ≥ 0 values leads to a limited density of the surface charge *q_e_* which can be moved:

(88) $$q_{e}\leq U\frac{{\text{εε}}_{0}}{d}=q_{C}$$

It can also be deduced from discussion above that the surface density of charge *q_e_*, which can be moved off by external voltage, depends on the initial location point ψ*_e_* of charge *q_e_* within the inter-electrode gap *d*, and on the applied voltage *U*, *i.e.*, on the steady-state charge *q_C_* on the electrode. Thereby, a pure Ramo’s regime is hardly possible for electrodes of a finite area, due to the necessity of the synchronous holding of τ*_Mq_*_,*e*_/τ*_TOF_*_,*e*_ = 1 and *C_Sq_* > 0. This Ramo’s regime can be approximately approached at large injected charge densities and low applied voltages. Just after the injection of a large charge *q_e_* > *q_C_*, which completely screens *q_C_* (terminates the field *q_C_*/εε_0_ due to *q_C_* < *q_e_*), charge *q_e_* can only disappear from the inter electrode space through a diffusion (with a carrier diffusion coefficient of *D*) process. The last process leads to a rather long current transient, extending within the time scale of τ*_D_* = 1*/*η_1_^2^*D* = *d*^2^*/*π^2^*D* [30,31,32,33]. Other peculiarities of the transforms of the injected charge domain drift regimes are in detail discussed in [17,20].

## 4. Impact of Drifting Charge Variation in Time

The current density depends on the charge position change rates *d*ψ*_kl_*/*dt*, if carrier capture can be ignored. In the general case, charges are dependent on time due to different effects. For instance, charges can be modified: *q_C_* = *C_g_*(*U* − *i*(*t*)*R_L_*) = *q_C_*_0_exp(*−t*/τ*_RC_*) due to voltage drop *i*(*t*)*R_L_* and delay (τ*_RC_*) within an external circuit; *q_e_* = *q_e_*_0_exp(−*t*/τ*_c_*_,*e*_) and *q_h_* = *q_h_*_0_exp(−*t*/τ*_c_*_,*h*_) due to carrier capture on traps in a dielectric material, respectively, *etc.* Thereby, the dependence on time of the bulk charge ρ *= q*/*d* can be represented as:

(89) $$\frac{1}{d}\frac{dq}{dt}\equiv \frac{d\rho }{dt}=-\gamma_{1}\rho -\gamma_{2}\rho^{2}-\gamma_{3}\rho^{3}-....$$

A coefficient of the linear relaxation γ_1_ can be ascribed to τ*_c_*,*_e_* and τ*_c_*,*_h_*, the carrier capture times, and even to the Maxwell dielectric relaxation time τ*_M_ =* εε_0_Ω related to material resistivity Ω, if the last is determined by the charges within the dielectric material (except the moving charge *q*). The squared term γ_2_ρ^2^ can be attributed to the current induced by the drifting charge *q*. This happens when carriers move within a plane of the surface domain. Then charge self-correlates its position ψ and rate *d*ψ/*dt* of position changes, evaluated in this consideration as τ*_Mq_ = d*εε_0_/μ*q =* 1*/*γ_2_ρ.

Depending on the important terms, involved within Equation (89), expressions describing the time dependent variations of a moving charge are obtained rather differently, as, for instance:

(90) $$q(t)=\{\begin{array}{c} \begin{array}{l} q_{0}\exp(-{\text{γ}}_{1}t)\text{ for }{\text{γ}}_{1}\neq 0\;{\text{γ}}_{m+1}=0 \end{array}\\ \begin{array}{l} \frac{q_{0}}{1+{\text{γ}}_{2}q_{0}t}\text{ for }{\text{γ}}_{1}=0\;{\text{γ}}_{2}\neq 0\;{\text{γ}}_{m+2}=0 \end{array}\\ \frac{q_{0}\exp(-{\text{γ}}_{1}t)}{1+\frac{{\text{γ}}_{2}}{{\text{γ}}_{1}}q_{0}(1-\exp(-{\text{γ}}_{1}t))}\text{ for }{\text{γ}}_{1}\neq 0\;{\text{γ}}_{2}\neq 0\;{\text{γ}}_{m+2}=0 \end{array}$$

Here, *q*_0_ = *q*(*t* = 0).

To describe current transients by including *q*(*t*), when drifting charge varies in time, the equations of type Equations (5) and (20) should be re-arranged as:

(91) $$i=S\frac{d}{dt}\sigma =S(q\frac{d}{dt}\psi +\psi \frac{d}{dt}q)=S(q\frac{d}{dt}\psi -(\gamma_{1}q+\gamma_{2}q^{2}+...)\psi )$$

This Equation (91) indicates that there appears a current (displacement) component *i_G_* = *−S* (γ_1_*q +* γ_2_*q*^2^ + *…*)ψ, additional to the Ramo’s (*i_R_* = *Sqd*ψ/*dt*) type drift (convection) current component. This happens due to τ*_Mq_*/τ*_TOF_* ≠ 1 even in the case of the ignored carrier capture. This additional current *i_a_*, necessary to compensate for the misbalance (τ*_Mq_*/τ*_TOF_* ≠ 1) of the action/reaction rates, can be written as:

(92) $$i_{a}=S\psi \frac{d}{dt}q$$

Then, the rate equation is expressed through the time dependent parameters τ*_Mq_*(*t*) and τ*_TOF_* (*t*, *U*, ψ) as:

(93) $$\frac{d}{dt}\psi -\frac{\psi }{\tau_{Mq,l}(t)}-\frac{1}{\tau_{TOF,l}(\psi ,t,U)}=0$$

Evaluation of carrier trapping within the insulating material during a monopolar drift of electrons is simpler. Then, the equation for the changes of surface charge on electrode should be rearranged as:

(94) $$\sigma (t)=U\frac{\varepsilon \varepsilon_{0}}{d}-q_{e}(t)(1-\psi )$$

The charge dependence on time for the simple traps can be expressed as:

(95) $$q_{e}(t)=q_{e0}\exp(-t/\tau_{C})$$

Then, the current is expressed as:

(96) $$i_{tr}(t)=S\frac{d\text{σ}}{dt}=S[\frac{q_{e}(t)}{{\text{τ}}_{C}}(1-\text{ψ}(t))+q_{e}(t)\frac{d\text{ψ}(t)}{dt}]$$

It can be noticed in Equation (96), that the complete current now contains *i_G_*, the charge induction type current, and *q_e_*(*t*)*Sd*ψ/*dt*, an inherent drift current component. Introducing a trapping dependent dielectric relaxation time as:

(97) $${\text{τ}}_{Mq,e,tr}(t)=\frac{{\text{εε}}_{0}}{{\text{μ}}_{e}(q_{e0}/d)\exp(-t/{\text{τ}}_{C})}={\text{τ}}_{Mq,0}\exp(t/{\text{τ}}_{C})\;$$

the equation for the rate of dimensionless position changes of the charge domain. It can be re-arranged as:

(98) $$\frac{d{\text{ψ}}_{e}}{dt}-\frac{\exp(-t/{\text{τ}}_{C})}{{\text{τ}}_{Mq,0}}{\text{ψ}}_{e}-\frac{1}{{\text{τ}}_{TOF}}=0$$

with ψ*_e_* = *X*_e_/*d* and the respective boundary conditions. While, τ*_Mq_*_,0_ is defined by Equation (79). Solution of Equations (92) and (98) can be expressed commonly [22]. Although, this Equation (98) is as usually solved numerically. The drift time *t_dr_* is then determined by using a boundary condition ψ(*t_dr_*) = 1, which leads to a transcendental equation, and it again should be solved numerically.

The drift current component can be ignored (∂ψ/∂*t* ≈ 0) and, consequently, the drift current component nearly disappears due to its small value, if the injected charge lifetime τ_C_, due to trapping, is the shortest one within a set of characteristic relaxation parameters. Then, Equation (96) can be simplified as:

(99) $$i_{tr}(t)=\frac{q_{e0}S\exp(-t/{\text{τ}}_{C})}{{\text{τ}}_{C}}\;$$

which describes the induction current, arisen due to the local changes of the injected *q_e_*_0_ charge. This can be understood as a difference of displacement currents, which arises due to the necessity to keep the external voltage invariable and to complete the circuit, when the induced charge at ψ_0_ ≠ 0 rapidly disappears. For ψ_0_ = 0, this displacement current component compensates for a conductivity current at the boundary electrode, to complete a circuit with capacitor considered.

**Figure 6:**
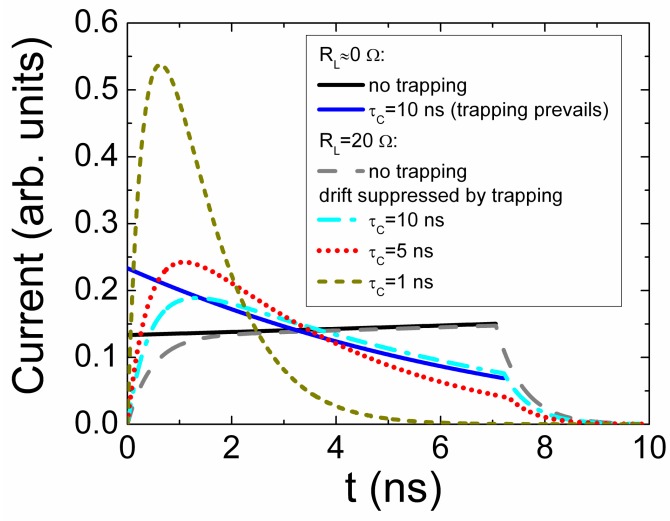
Current transients during monopolar drift of small charge at different carrier capture lifetime τ*_C_* values. The solid curves are calculated using analytical expressions (Equations (92)–(98)), while the dashed curves are obtained including the RC of the signal recording circuit.

Evaluation of other parameters (e.g., *t_dr_*) of the transients determined by the injected charge drift and trapping becomes even more complicated, and it can only be implemented by numerical methods. For the prevalence of trapping processes, no articulated features of domain drift response can be separated, and only the relaxation-type shape following the charge domain injection peak can be observable within the current transient (Figure 6). Trapping (Figure 6) leads to simultaneous changes in the moving charge density and velocity field. Then, current density variations in time acquire a relaxation curve shape. Nevertheless, analysis of the experimental current pulse shapes using the modelled transients enables ones to discriminate the prevailing processes of either the injected charge drift or carrier capture and to evaluate the parameters of these processes.

## 5. Current Transient Changes Determined by a Signal Recording Circuit

A signal registration circuit (namely, a load resistor) inevitably transforms the transient shape due to the fact that the voltage on the detector changes depending on the current value within the external circuit. In more general cases, the transients are described by solutions of the differential equation with variable coefficients, derived as:

(100) $$\begin{array}{l} \frac{\text{σ}(t)}{C_{Sq}(t)}+iR_{L}=U\text{ with }i=S\frac{d\text{σ}}{dt}\\ \frac{1}{C_{Sq}}\frac{d\text{σ}(t)}{dt}-\frac{\text{σ}(t)}{C_{Sq}}\frac{dC_{Sq}(t)}{C_{Sq}dt}+\frac{di}{dt}R_{L}=0\\ \text{with }U_{CR}=U-iR_{L}\text{ and }U_{CR}=\frac{\text{σ}(t)}{C_{Sq}} \end{array}$$

This leads to a non-linear differential equation:

(101) $$\frac{di}{dt}+(\frac{d\ln SC_{Sq}(t)}{dt}+\frac{1}{R_{L}SC_{Sq}(t)})i=\frac{U}{R_{L}}\frac{d\ln SC_{Sq}(t)}{dt}$$

which should be solved by using the initial conditions:

(102) $$\begin{array}{l} i_{0}(t=0)=0\\ SC_{Sq}(t=0,q_{e}=0)=C_{0} \end{array}$$

for the ascending component within a current transient, and:

(103) $$\begin{array}{l} i_{0,r}(t=t_{dr})=j(t=t_{dr})S=i(t_{dr})\\ SC_{Sq,r}(t=t_{dr},{\text{ψ}}_{e}=1)=C_{0} \end{array}$$

for the relaxation stage of a current pulse, respectively.

The changes of the system dynamic capacitance determine the initial delay and final stage (relaxation) components within simulated transients (Figure 6). These components are inevitable in experimentally registered current transients ascribed to the charge drift. These components should be included into an evaluation of charge collection efficiency measurements. Depending on the geometrical capacitance (*C_g_*) and load resistor (*R*_L_), the current pulses are significantly modified.

## 6. Impact of Charge Multiplication Effects

### 6.1. Analysis of Multiplication Factor

Let’s consider the multiplication factor *M*(*x*), similar to well-known model [37,38], where a single carrier pair *M*(*x*) *=* 1 is generated at location *x* within device depth *w* and the multiplication of additional pairs appears in the interval 0 ≤ *x*' < *x* < *x*'' ≤ *w* due to impact ionization with coefficients of α and β(in cm^−1^) for electrons and holes, respectively. Let’s assume, that holes move a distance *x*' to the left relative to initial location *x* while electrons drift to the right from *x*, using a circuit similar to that analyzed in [17,20]. Such a drift with multiplication is described by a differential equation for different segments of hole and electron drift as:

(104) $$\frac{dM(x\prime )}{dx\prime }=\{\begin{array}{c} -\text{β}M(x\prime )\text{ for }0\leq x\prime <x\\ 0\text{ for }x\prime \equiv x\\ \alpha M(x\prime )\text{ for }x>x\prime \geq w \end{array}$$

including the directions of motion. This equation in segments should satisfy the boundary (initial, *t* = 0) condition:

(105) M(x′,x)=1 for x′=x

Solution of Equation (104) with boundary condition Equation (105) leads to expressions for each segment:

(106) $$M(x,x\prime ,x\prime \prime )=\{\begin{array}{c} \exp[\text{β}(x-x\prime )]\text{ for }0\leq x\prime <x\\ 1\text{ for }x\prime =x=x\prime \prime \\ \exp[\text{α}(x\prime \prime -x)]\text{ for }x>x\prime \prime \geq w \end{array}$$

Integrating Equation (104) over the entire range of 0< *x* < *w* (for a fixed initial location *x* of a pair)*,* one obtains:

(107) $$M(x)=1+\int_{0}^{x}\beta M(x\prime )d(-x\prime )+\int_{x}^{w}\alpha M(x\prime \prime )d(x\prime \prime )$$

This equation coincides with that considered within wide-spread model [37,38,39,40]. Inserting solutions Equation (106) into Equation (107) for the relevant segments, Equation (107) is rearranged as:

(108) $$M(x)=e^{\beta x}+e^{\alpha (w-x)}-1=2e^{\frac{(\beta -\alpha )x}{2}}e^{\frac{\alpha w}{2}}\cosh[\frac{(\alpha +\beta )x-\alpha w}{2}]-1$$

with boundary values of *M*(0) = exp(α*w*), *M*(*w*) = exp(β*w*) and *M*(*x*) = 1. The obtained Equation (108) indicates that a finite multiplication factor is only possible for the finite width *w*. The total multiplication factor is evaluated (with initial condition in Equation (105)) by:

(109) $$<M(x)>{|}_{\alpha ,\beta }=\int_{0}^{w}\alpha M(x){|}_{\beta =0}dx+\int_{0}^{w}\beta M(x){|}_{\alpha =0}dx+1=e^{\alpha w}+e^{\beta w}-1$$

The obtained Equation (108) indicates that a finite multiplication factor is only possible for the finite width *w*. The averaged multiplication factor over width *w* is then evaluated as:

(110) $$<M(x)>{|}_{w}=\frac{1}{w}\int_{0}^{w}M(x)dx=\frac{1}{\beta w}[e^{\beta w}+\frac{\beta }{\alpha }(e^{\alpha w}-1)-(1+\beta w)]$$

Equation (109) leads also to the normalization relation <*M*(*x*)> = 1 for the simultaneously fulfilled conditions of α*w* << 1 and β*w* << 1. Alternatively, another normalization (to the initial, *t* = 0, condition *M*(*x*)*|_t_*_=0;*x'*=*x*_ = 1) relation can be introduced by:

(111) $$<M(x)>{|}_{\alpha =\beta }=\int_{0}^{w}\alpha M(x)dx=[2(e^{\alpha w}-1\}-\alpha w]{|}_{\alpha w<<1}≅\alpha w{|}_{\alpha w\to 1}\Rightarrow 1$$

This Equation (111) again coincides with the normalization condition [37] for the impact ionization. Although, value of <*M*(*x*)*>|_αw_*_=1,α=β_ ≥ 2.4 is obtained for α*w* = 1 at α = β.

The pure bipolar drift and multiplication lead to charge collection at electrodes of a set of generations (cascades) of carriers appeared due to impact ionization. Equation (108) represents multiplication obtained for the first generation *M*(*x*, 1) of multiplied carriers determined by drift of holes and electrons in opposite directions from the initial point *x* within external field, sufficient for impact ionization. Only single type of carriers (holes or electrons) is collected at relevant electrode. Other counter-partners (for instance, electrons, if holes are collected at *x* = 0) of each multiplied pair are separated by diffusion-drift and move from this electrode and initiate the next generation (cascade) of carrier multiplication. For a symmetric pure bipolar process (α = β, *x = x_h_* = (*w* − *x_h_*)*_e_*, *v_h_* = *v_e_*), electrons with initial (at *x* = 0) quantity exp(β*x*) − 1 generate pairs (within transit time τ*_tr_ = x_h_*/*v_e_*), and the enhanced quantity of pairs reaches (at *x'* = *x*) value *N*_2,*x*_ = *∫*_0_*^x^*α(*e*^β*x*'^ − 1)*dx'*. The holes of these *N*_2,*x*_ pairs during the time *t* interval τ*_tr_* < *t ≤* 2τ*_tr_* generate the second set of collected charge *M_h_*(*x*, 2) = *∫*_0_*^x^*β*N*_2,*x*_*dx'*. By considering the same process within (*w* − *x_h_*)*_e_* for electron quantity multiplication, the same *M_e_*(*x*, 2) value is obtained. The total quantity of the second generation carriers is *M*(*x*, 2) = 2[e^α*x*^ − 1 − α*x* − (α*x*)^2^/2]. By continuing analysis of the next generations of carriers (multiplication cascades) the multiplication factor for each generation *n* is derived as:

(112) $$M(x,n)=2[e^{\alpha x}-\sum_{m=0}^{2n-2}\frac{{\left(\alpha x\right)}^{m}}{m!}]+1$$

for the symmetric pure bipolar drift and multiplication process (α = β*, x = x_h_* = (*w* − *x_h_*)*_e_*, *v_h_* = *v_e_*). The cumbersome expressions can be obtained for asymmetric bipolar drift (τ*_tr_*_,*b*_ = *x_h_*/*v_h_* =(*w* − *x_h_*)/*v_e_*) with properly adjusted coefficients α and β*.* In general case of mixed (bipolar proceeded by monopolar) drift regime, the re-calibration of charge on electrodes should be included [17,20], as discussed in Section 2.

### 6.2. Models of Current Transients with Charge Multiplication

The finite multiplication factor of value *M*(*x*) > 2 stimulates search for regimes of current signal formation with *αw* ≥ 1, to get the efficient internal gain. In particle detectors, reduction of carrier lifetime due to radiation defects should be compensated (at least) by proper multiplication *M*(*w*). Both parameters, carrier pair multiplication factor *M*(*x*) and carrier lifetime τ, lead to a change of carrier drift velocity *v = w*(*d*ψ/*dt*) with variation of the dimensionless position ψ = *x*/*w* due to carrier drift in Section 2. It can be deduced from Equation (8) that drift velocity depends on dimensionless position ψ of moving charge (due to a change of Coulomb force with distance). Actually, due to different mobilities of holes and electrons, different acting electric fields and velocities appear for holes and electrons. For the pure bipolar drift (Equations (106)–(112)), the synchronous arrival of electrons and holes to opposite electrodes is held by making drift of electrons and holes with invariable velocity. Thus, to keep the same transit time τ*_w_*:

(113) $$\tau_{w}=\frac{x}{\left(w\left(\frac{d\psi_{h}}{dt}\right)\right)}=\frac{w-x}{\left(w\left(\frac{d\psi_{e}}{dt}\right)\right)}$$

the coordinate and field dependent ratios of the impact ionization coefficients α(*E*, *x*)/β(*E*, *x*) = *x*/(*w* − *x*) should appear. Really, an impact ionization event would appear for simultaneous matching of energy and momentum conservation [41]:

(114) *mv*^2^/2 = 2.3ε*_G_* + 0.7 (eV)

(115) *mv = hk*(ε) + *h*κ*_ph_*

The expressions in the latter parenthesis are written using relation for phonon assisted process in material with forbidden energy gap ε*_G_* defined in eV [41] and drift momentum related to quasi-momentum and the energy band spectrum *k*(ε). Actually, to satisfy energy and momentum conservation for varied acting electric field *E*(ψ) the spectrum κ*_ph_* of the needed phonon filled states should be rather wide. This depends considerably on lattice temperature. For the mixed drift regime, when electrons and holes arrive to electrodes at different time instants, consideration becomes more complicated [17,20]. Assuming here that conditions for pure bipolar drift is held, every carrier pair carries an elementary charge under arrival to electrodes, and there appear the collected charge:

(116) *Q_M_* = *e<M|*_α,β_*>*

and current:

(117) *i* = *Q_M_*/τ*_tr_*

Variations of the acting electric field *E*(ψ(*t*)) in time and as a function of dimensionless position ψ(*t*) can be determined using the dynamic approach [17,18,19,20].

The dynamic model can be applied for analysis of cascade processes with multiplication and carrier capture. In addition to vacuum-type detectors, scattering of the injected carriers is inevitable in solid media, which fills the inter-electrode gap. Impact ionisation can also be considered as a scattering process with varied carrier pair quantity. To solve the dynamic problem of drift of the injected charge domain, parameters determined at steady-state conditions, such as carrier mobility *etc.*, can be employed. Alternatively, the dynamics of space and time dependent variations of the drift velocity can be considered based on solutions obtained for vacuum capacitor. There, a free mean path λ can be assumed to be a characteristic drift distance, *i.e.*, *w* = λ and with characteristic relaxation times τ*_TOF_*_,λ_, τ*_M_*_,λ_. The free mean path λ can be estimated statistically and by using Chynoweth’s law [38,39,40] for the coefficients of impact ionization, as:

(118) λ(*E*) = α_∞_^−1^exp(*E_thr_*/*E*)

which leads to shortening of λ with enhancement of acting field *E.* The acting voltage over a free path before impact ionization, as *U*_λ_, is defined by *U_λ_* = ∫_X_^X+λ^*E*(*t*, *X*_λ_)*dX*_λ_*.* The current transient then is comprised of fragments of the injected charge drift over a set of η *= w*/λ of free paths. Really, the scattering process with the shortest free mean path λ*_si_* should be assumed as the prevailing one:

(119) 1/λ = 1/λ*_si_* + Σ*_i_*_–1_1/λ*_i_* ≈ 1/λ*_si_*

if different scattering processes can be assumed as acting in parallel and being linear (without interaction). Similarly, carrier capture process, which determines the shortest carrier lifetime τ*_si_*, will dominate:

(120) 1/τ = 1/τ*_si_* + Σ*_i_*_–1_1/τ*_i_* ≈ 1/τ*_si_*

### 6.3. Simulation Assumptions and Algorithms

To evaluate the possibility and the main regimes to compensate recombination through radiation defects (with τ*_si_* = τ*_rd_* by impact ionization *M*(*x*, *n*) and λ*_si_*), let consider simplified assumptions: (I) the applied voltage *U* is sufficient to initiate the impact ionization determined by inequality (*m*/2)[{*e*τ*_tr_*_,λ_(*d*/λ)/*m*}(*U*/*d*)]^2^ *>* 2.3ε*_G_* + 0.7 (in eV); (II) pure bipolar drift of carriers takes place, where single pair carries an elementary charge of electron under simultaneous arrival of hole and electron to opposite electrodes, separated by a distance *d*, *i.e.*,:

(121) $$\frac{e_{h}}{d}\int_{x}^{0}dx\prime +\frac{e_{e}}{d}\int_{x}^{d}dx\prime \prime =e(-\frac{x}{d})+(-e)(\frac{d-x}{d})=-e$$

and the total current ascribed to a single pair drift is obtained:

(122) $$|i_{\;eh}|=\frac{e}{\tau_{tr,e}}\text{ with }\tau_{tr,e}=\tau_{tr,h}$$

Equation (122) indicates that transit time varies with *x* due to ∆*x_h_ = v_h_*τ*_tr_*_,*h*_ ≠ ∆*x_e_ = v_e_*τ*_tr_*_,*e*_ at τ*_tr_*_,*h*_ = τ*_tr_*_,*e*_ if drift velocities *v_h_* ≠ *v_e_* of holes and electrons, respectively, are different. The current generated by pure bipolar drift always exceeds that of the monopolar one due to the shorter transit time, irrespective that the carried charge is the same; (III) the pure bipolar drift regime determines the invariable drift velocity [17], independent of instantaneous position of drifting carriers; (IV) the pure bipolar drift regime justifies derivation of Equations (104)–(123) (with initial bipolar charge injection) and application of multiplication factor *M*(*x*, *n*) expressions. Current variations in time due to collection of each generation *n* (cascade) of multiplied carrier pairs is obtained by assuming that *x_h_* = *v_h_t* for (*n* − 1)τ*_tr_<t* ≤ *n*τ*_tr_*:

(123) $$i(t,n)=\frac{qSM(t_{n},n)}{\tau_{tr}}M(t_{n-1}-\tau_{tr}=t_{n}=0,n-1)\exp[-(\frac{1}{\tau }+\frac{1}{\tau_{Dn}})t_{n}]$$

and using expressions of *M*(*t_n_*, *n*) described by Equation (112). Here, *qS* = *eR* is the charge carried by the initially (at *x*, *t* = 0) injected *R* pairs, *t = t_n_* + Σ*_p_*_=0_*^n^*^−1^*p*τ*_tr_* is the running time*.* The pure bipolar drift regime can be held by varied instantaneous values of coefficients α and β of the impact ionization dependent on acting field and location of drifting charges. The ambipolar diffusion of narrow injected carrier pair domain is included using time dependent solutions of diffusion problem in finite thickness area [30,31,32,33]. Ambipolar diffusion is there characterized by the decay time τ*_Dn_* = 1/*D*ζ*_n_*^2^ with spatial frequency ζ*_n_* of the main decay mode ascribed to a spatial width δ of the injected domain and including its broadening during each *t_n_* of multiplication cascade*.*

Using assumptions (I)–(IV) and a dynamic model based on Shockley-Ramo’s theorem [8,9], the simplified simulation algorithm for emulation of impact ionization attributed to the first generation carrier pairs can be made employing equal free mean paths λ*_ii_*, attributed to the impact ionization, at the end of which quantity of pairs is nearly doubled with *m* ≤ *M*(*x*) *>* |_λ*ii*_, leading to η *= d*/λ*_ii_* segments of double multiplication of pairs. Then, the transit time τ*_tr_*_,λ*ii*_ is estimated for each segment of double multiplication, including of Ramo’s current [8] dependence on surface carrier density and location of each segment within inter-electrode gap. The complete transit (with multiplication) time τ*_tr_*_,Σ,*e*_ is a sum τ*_tr_*_,Σ,*e*_ = Σ_λ*ii*_τ*_tr_*_,λ*ii*,*e*_ of transit times over drift segments for either electrons or holes. Competition of recombination (τ) and multiplication *m* = *M*(*t*, *n*) in Equation (112) is accounted for in modification of drift velocity within each segment by a sum of quantities (−τ*_tr_*_,λ*ii*_/τ) + *M*(τ*_tr_*_,λ*ii*_,1) ≥ 1*.* Additionally, dependence of acting field dependence on the amount of drifting charge (*eR*η_e_*<M*(*x*)*>|*_λ*ii*_) with approach of *x* (or (*d* − *x*)) to electrode is included, according to dynamic model [17,18,19,20], in other words, shortening of τ*_Mq_* with increase of the amount of drifting charge is taken into account.

The multiplication process is limited by the amount of multiplied drifting charge: *eM*(*t*, *n*) ≤ *Q_B_ = CU*, *i.e.*, it cannot exceed the charge *Q_B_* supplied by battery of voltage *U* to a device of capacitance *C.* For large multiplication, the increased charge *eM*(*t*, *n*) ≈ *Q_B_* of free drifting carriers will screen (via electrostatic induction) the externally created charge *Q_B_* on electrodes. Then, the current changes are determined by charge collected at electrodes, brought by diffusion.

**Figure 7:**
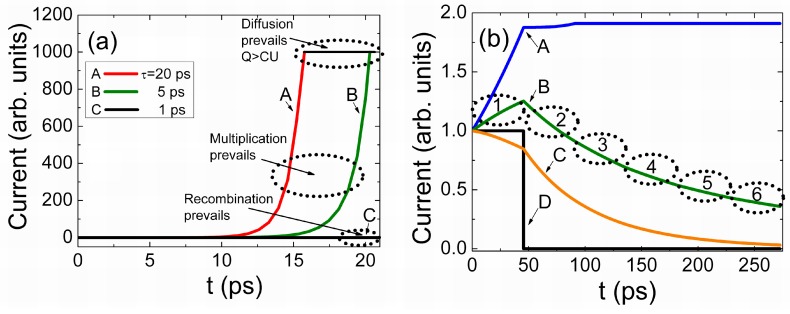
Comparison of simulated current transients for capacitor-type detector of GaN. (**a**) The initial component (of the first generation multiplied carrier pairs) of current transients, simulated using the described (in Section 6.2) dynamic model with parameters: <*M*(*x*, 1)>_λ_ = 2, *d* = 2 μm, *U* = 10 V at initial amount of injected pairs *R* = 10^5^ and varying recombination lifetime: A—τ = 20 ps, B—5 ps, C—1 ps; (**b**) Current transients including the higher generations of multiplied carrier pairs simulated using Equations (113) and (123) with parameters *D* = 2 cm^2^/s, δ = 0.5 μm, *d* = 5 μm, *v* = 10^6^ cm/s, α = β = 8,000 cm^−1^ and carrier lifetimes A—τ *=* τ*_D_* = ∞, B—τ = 1 ns, C—τ = 100 ps; D—the current transient for pure bipolar drift without multiplication. Numbers indicate the cascades of transits.

Variations of current transients in capacitor type detector, simulated using the discussed assumptions and dynamic models with a set of parameters (exploited within software package Synopsys TCAD Sentaurus) for GaN filled capacitor-type detector of inter-electrode gap *d* = 2–5 μm, are illustrated in Figure 7.

### 6.3. Simulation of Current Transients Including Multiplication Effect by TCAD

#### 6.3.1. Simulation Approach and Parameters

For comparison of dynamic and quasi-steady-state models, the standard simulations of current transients for PIN diodes have alternatively been performed by employing a drift-diffusion model implemented using the software package Synopsys TCAD Sentaurus. The current transients caused by an injected bipolar charge domain have been simulated accounting for the drift-diffusion, carrier capture/recombination and charge multiplication processes.

The PIN diode structure of the 1 µm width N^+^ and P^+^ regions with doping density of 10^20^ cm^−3^ at the contacts of abrupt junctions with a 5 µm thick drift layer of intrinsic resistivity material was chosen for consideration. The junction area of *S* = 7.85∙10^−5^ cm^2^ was assumed. Particle detection has been emulated using photo-excitation with GaN absorption coefficient at λ = 473 nm wavelength. Calculations were made for injection of an excess carrier domain at different locations *x*_0_ within active large resistivity volume of a PIN diode. A width δ = 0.1 µm excitation beam of a strip shape was assumed. Excitation 2 ps pulse of intensity 10^4^ W/cm^2^ was chosen for simulations. Emulation of the impact ionization processes was performed with parameters of GaN, namely, with impact ionization coefficient *α* at infinite electric field α_∞__,*e*_ = 2.9 × 10^8^ cm^−1^ and threshold electric field value of *E_thr_*_,*e*_ = 3.4 × 10^7^ V/cm for electrons as well as α_∞__,*h*_ = 5.41 × 10^6^ cm^−1^ and *E_thr_*_,*h*_ = 1.96 × 10^7^ V/cm for holes, respectively. Here, validity of the Chynoweth’s law [38,39,40] is assumed. Values of saturation velocity of *v*_e,s_ = 1.8 × 10^7^ cm/s for electrons and of *v*_h,s_ = 10^7^ cm/s for holes, respectively have been chosen according to parameter set enclosed within software package Synopsys TCAD Sentaurus. For the low field regime in the intrinsic material layer, values of the mobility have been assumed at room temperature (T = 300 K), as μ*_e_* = 1500 cm^2^/Vs for electrons and μ*_h_* = 100 cm^2^/Vs for holes, respectively.

The standard simulation procedures have been performed by using algorithms based on a drift-diffusion model including a solution of Poisson’s equation for electrostatic potential and continuity equations for electrons and holes involved within Synopsys TCAD Sentaurus software platform. There, charge neutrality and equilibrium state conditions are assumed at ohmic contacts. Simulations were made assuming a simple circuit consisting of a constant voltage source connected in series with the diode and load resistance *R*_L_ of values *R*_L_ ≈ 0 and 50 Ohm. The quasi steady-state approach is traditionally exploited with drift velocity determined by steady-state field distribution and its dependence on field strength represented parametrically, based on the steady-state characteristics. The impact ionization has been accounted for via generation rate *G* = α_e_*nv*_e,s_ + α_h_*pv*_h,s_ of electrons (*n*) and holes (*p*), respectively. Shockley–Read–Hall (SRH) type recombination has been assumed to be prevailing with doping dependent microscopic capture lifetimes for electrons and holes. These values microscopic capture lifetimes have been varied in order to evaluate opportunity of the suppression of carrier capture by charge multiplication at sufficiently large applied external voltages.

#### 6.3.2. Simulation Results

**Figure 8:**
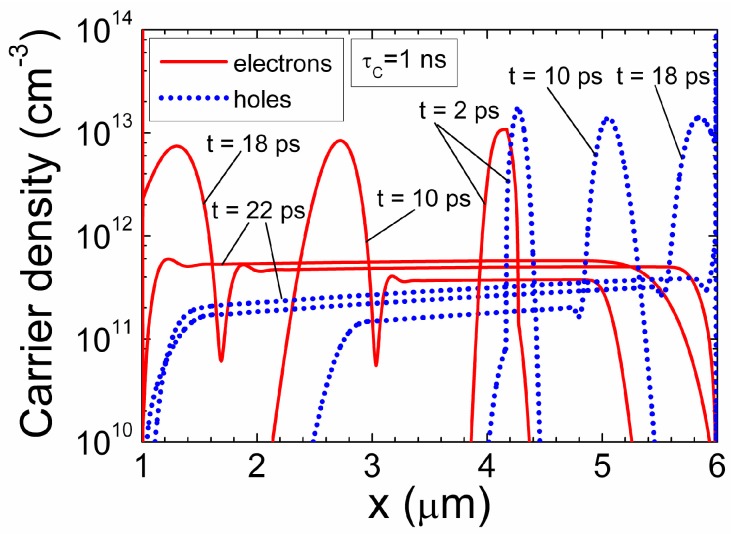
TCAD simulated excess carrier density depth profiles within diode active layer of intrinsic GaN material during evolution of separated carrier domains, which are initially injected at *x*_0_ = 4.2 µm relative to the N^+^ contact. Here, the external parameters of *U* = 1325 V and *R_L_* = 0 are used.

The TCAD simulated profiles of depth distribution of excess carrier density at increasing time instants are illustrated in Figure 8. These profiles are obtained by standard TCAD solving procedures including carrier drift, diffusion, capture and multiplication. The last process is inherent for large voltages in narrow active layer device.

The spatially separated peaks (which can be attributed to the respective charge domains) of excess electrons and holes can be deduced from these profiles. The widened tails of carrier density behind the peaks can also be observed, in Figure 8. However, in contrast with intuition concerning the increase of the strength of acting field, peak density values decrease with approach of carrier domains to the attracting field electrodes for holes and electron, respectively. Complementarily, the dimples of the amplitudes increasing with time appear just after initial domains. The tail ranges of intersecting plateaus of excess electrons and holes can be additionally revealed within simulated profiles of the carrier concentration instantaneous distribution over the inter-electrode gap, Figure 8. These plateaus imply significance of Dember internal electric field, appeared due to ambipolar diffusion, relative to external source voltage field. The ambipolar diffusion and recombination of carriers prevail within these of intersecting ranges. The front profiles of drifting domains, characterized by charge density peaks, are influenced by carrier capture and multiplication processes.

**Figure 9:**
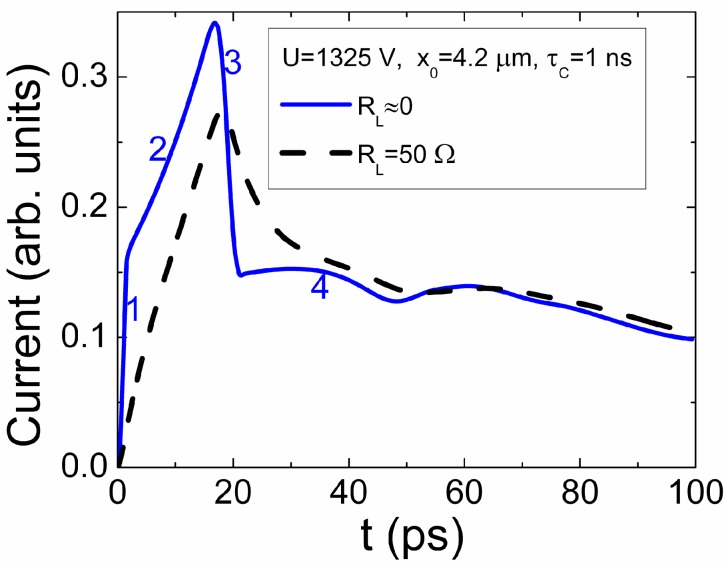
Comparison of the diode current transients simulated for the same charge multiplication conditions with different values of load resistance *R*_L_:*R*_L_ ≈ 0 Ohm (solid line) and *R*_L_ = 50 Ohm (dashed line). Numbers indicate different stages within a current transient.

The simulated evolution of carrier density distribution profiles determines duration and shape of TCAD modelled current transients, Figure 9. The TCAD simulated current pulses, using different load resistances within external circuit loop, are illustrated in Figure 9, by considering the same drift-diffusion-multiplication-recombination processes and parameters. It can be deduced from Figure 9 that at negligible voltage sharing between device and load (*R*_L_ ≈ 0), fragments of current changes (solid line) in time ascribed to charge domain injection (1) and its drift with multiplication (2, 4) can be resolved. There, a discontinuity (3) of current value appears just after primary charge transit time. This discontinuity can naively be attributed to the charge-mass transport to electrode. Existence of significant external load (dash line in Figure 9), and its action as an RC chain with rather short time constant (*R*_L_*C*_d_ ≈ 7 ps), comparable with duration of drift processes, may hide the essential phases in formation of current pulse (dash line transient). The multiplication process, proceeded by the higher generations of excess carriers, determines the delay of charge collection.

The simulated evolution of injected charge domains and of carrier density depth-distribution profiles can be qualitatively explained by prevailing of injected domain separation and drift under large external voltage on electrodes. The multiplication of carriers can be only implied through widening of peaks. *i.e.*, through increase of volume of the enhanced carrier density while its peak amplitudes are reduced with approach to electrodes. Such type evolution of multiplication process (Figure 8) is different from that (Figure 7) deduced in dynamic model where the multiplication factor, the electric field and the drift velocity considerably vary with increase of carrier amount. The current changes in time are continuous for transients simulated using the dynamic model, as the surface charge on electrodes varies consequently due to the higher generations of the multiplicated carriers and the displacement field caused by their drift. In steady-state regime, which is used in drift-diffusion approach exploited in TCAD, charge on electrodes is assumed to be invariable at constant potential. Therefore, the image charge supplied by external voltage source is completely ignored in drift-diffusion approach, in contrast to Shockley-Ramo’s theorem. Thus, the steady-state regime can be supported at invariable drift velocity (the main assumption in solution of Boltzmann equation [10]). Then, a semiconductor device acts as time-space varied resistance (in quasi-steady-state simulation approach), dependent on excess carrier density instantaneous distribution. There, the charge induction phenomenon (appearance of Ramo’s current within external circuit loop) is ignored. As a result, the dimples of the amplitudes increasing with time (Figure 8) and a discontinuity of current value (3 in Figure 9) appear when using the quasi-steady-state simulation approach in TCAD.

## 7. Discussion

The dynamics of space and time dependent variations of the drift velocity with scattering in dielectric filled capacitor can be considered, based on solutions obtained for a vacuum capacitor. Changes of regimes for a small charge drift can only be described approximately, as a one-dimensional charge momentum is not persistent, and retardation and magnetic field effects should be considered in general case [23]. For a high density of carriers within the induced charge domain, the ambipolar diffusion of the induced domain becomes dominant in the formation of the injected charge current pulse, when charge on electrodes is screened by the injected charge. Presence of carrier trapping considerably modifies the shape of the current pulse determined by the injected charge domain drift. This leads to an additional current component and to transformation of the drift velocity field. The impact of the dynamic capacitance and load resistance in the formation of drift current transients is important when delay appears due to long RC of the external circuit. The synchronous carrier drift and trapping lead to a vast variety of possible current pulse waveforms. Nevertheless, the analytical solutions obtained can be applied for consideration of the drift current transients in capacitor type detectors, based on wide band-gap materials.

The simplified models [17,18,19,20], based on Ramo’s expression for the drift current, are attractive as they provide a simple analytical description of the detector signals. However, the analytical expressions can only be obtained for the simplest approximations. The analytical form of the correlated drift (Ramo’s-type) current for the junction type detectors is only applicable for a primary estimation of a transient shape. Different regimes in the formation of the pulsed response of detectors can appear in a real measurement technology. The time-dependent variations of the current transients may be determined by the injected charge domain dissipation through the domain drift, dielectric relaxation (due to media polarization effects), through carrier capture and thermal release processes in the traps containing material, via ambipolar diffusion processes. Several specific aspects of these phenomena have been discussed above. The partial and full depletion regimes have been analyzed. It has been shown, that, in junction containing detector, the drift time of the rather small density injected surface charge domain is shortened relatively to that of the capacitor-like detectors when a proper frame of reference (for comparison) is accepted and characteristic relaxation times are matched. The description of the large injected charge drift current pulse shape in a finite area detector is coincident with that derived for the correlated drift (Ramo’s-type) expressions. However, the injected charge drift (ICD) current for the small injected charge and partial depletion regimes lead to deviations from the Ramo’s expressions. The analysis of the drift velocity field revealed the current increase within a vertex of the current pulse, for the monopolar drift regime. It has been shown, that presence of carrier traps considerably modifies the shape of the ICD current. For the extremely large density of the injected charge *q* > *C_g_U*, the ambipolar diffusion of the injected carriers may become dominant in formation of the injected charge current pulse. It has been illustrated, that synchronous action of carrier drift, trapping, generation and diffusion lead to a vast variety of possible current pulse waveforms. The discussed models and revealed features have been applied for analysis of charge multiplication processes.

The simulated current evolution in time during the first generation segment of multiplied carriers is illustrated in Figure 7a varying values of τ of recombination lifetime. For rather long recombination lifetime, increase of current is the fastest. There appears a rather long range of nearly linear increase of current. The charge amount increases with time (due to multiplication caused by approach of drifting charge to electrode) and determines enhancement of acting field according to Coulomb law. These factors, —the nearly exponential increase of carrier quantity due to multiplication and of their velocity, lead to a sharp increase of current with time, —the Geiger regime appears. However, for large multiplication, the increased charge of free drifting carriers will screen the externally created charge (due to applied voltage) on electrodes. The current value limited by (screening) diffusion is sketched by a horizontal line in Figure 7a.

The current transients consisting of a set of six generations (cascades denoted by dot circles and Roman ciphers) in multiplication sequence can be revealed in Figure 7b. The largest current appears for the first generation pairs (which transit time coincides with transit time of a counter-partner, *e* or *h*, without multiplication) due to the shortest transit time. Multiplied pairs of the second and the higher generations (Figure 7b) run the longer paths than those for the first generation ones and consequently appear at the later instants of running time *t*. These pairs are responsible for the decreasing current variation in time after the current peak formed by the first generation pairs. The recombination consequently makes the most important influence by reducing current values within the rearward phase of a current pulse due to increase of transit time (exp(−*t*_2_/τ) > exp(−*t*_3_/τ)*>…*)*.* Nevertheless, multiplication processes determine the rather long tail of current decrease (relaxation) after the peak of current caused by first generation carrier transit. Reduction of carrier recombination lifetime significantly shortens this relaxation component in current transient (Figure 7b). The current pulse of the multiplied charge drift appears to be significantly longer than the charge drift current without multiplication, Figure 7b (for comparison shown this nearly square-shape pulse simulated at applied voltage slightly below the multiplication threshold). The multiplication regime enables ones to increase significantly the collected charge relative to that of the current integral (area of a square-shape pulse) determined by pure bipolar drift without multiplication. Moreover, the delayed relaxation of current pulse over multiplication processes of higher generations overwhelms the time scale from picoseconds to nanoseconds. It is a proper time range in usage of particle detectors in measurements with shaping time of several nanoseconds. The waveforms of current transients are additionally dependent on the measurement circuit elements, due to voltage drop *i*(*t*)*r_L_* within load resistor and current rise delay (τ*_rC_*) within an external circuit.

## 8. Conclusions

A vast variety of regimes in the formation of the pulsed response of detectors can be implemented in real measurement and particle detection technology. Time-dependent variations of current may be determined by externally injected charge domain dissipation through domain drift and dielectric relaxation due to media polarization effects, through carrier capture in traps containing material, and via diffusion processes.

The analytical expressions can only be easily obtained for the simplest capacitor-type detector. Application of the simplified models presented is additionally limited by other factors. A principal limitation leads to threshold values of the acquired drift velocity that should be significantly less than those of the electric field (light) propagation velocity in the material under consideration, to ensure the validity of the electrostatic approach. This condition excludes the possibility to detect primary charged particles moving at relativistic velocities within the inter-electrode space. Values of the highest external voltages are also restricted by the necessity to exclude repeated and non-linear drift processes (as the photo-electric gain moderated by carrier trapping/thermal release, the avalanche processes of impact ionization or the Pool-Frenkel effect) in the application of the proposed models. The thermal generation (thermal release of excess carriers from traps) during charge domain drift complicates the equation for drift velocity temporal changes [17,20]. The diffusion of charge carriers might play an important role in the outspread of excess carriers and the appearance of the diffusion field which also determines the formation of the finite width of the injected quasi-neutral domain.

The dynamic models concerned with the formation of induced charge pulsed currents have been analyzed in capacitor-like detectors including multiplication effects. The derived analytical expressions can be applied for consideration of the drift current transients in capacitor type detectors, based on wide band-gap materials. It has been shown that internal gain is achievable in capacitor–type detectors based on GaN epi-layers. For a high density of carriers within the injected charge domain, the ambipolar diffusion of the induced domain becomes dominant in the formation of the injected charge current pulse, when charge on electrodes is screened by the injected charge. Presence of impact ionization and of carrier trapping considerably modifies the shape of the current pulse determined by the injected charge domain drift. This leads to modifications of the drift velocity field. The impact of the dynamic capacitance and load resistance in the formation of drift current transients is important [17,20] when delay appears due to long *r_L_C* of the external circuit and voltage sharing between load resistor and device under test. The similar qualitative evolution of current transients for GaN capacitor-type and PIN diode detectors has been obtained for multiplication regime.

The internal gain obtained by multiplication regime via impact ionization can be employed to suppress the degradation of detectors due to introduced radiation defects under large fluences of hadron beam irradiations. The technological parameters of the detector active layer width, geometrical capacitance and applied voltage should be adjusted to achieve suitable regimes for the internal gain and relevant pulse durations.

## References

[1] Martini M., Ottaviani G. (1969). Ramo’s theorem and the energy balance equations in evaluating the current pulse from semiconductor detectors. Nucl. Instrum. Methods Phys. Res. A.

[2] Cavalleri G., Gatti E., Fabri G., Svelto S. (1971). Extension of Ramo’s theorem as applied to induced charge in semiconductor detectors. Nucl. Instrum. Methods Phys. Res. A.

[3] De Visschere P. (1990). The validity of Ramo’s theorem. Solid State Electron..

[4] Eremin V., Strokan N., Verbitskaya E., Li Z. (1996). Development of transient current and charge techniques for the measurement of effective net concentration of ionized charges (*N*_eff_) in the space charge region of p-n junction detectors. Nucl. Instrum. Methods Phys. Res. A.

[5] Gatti E., Geraci A. (2004). Considerations about Ramo’s theorem extension to conductor media with variable dielectric constant. Nucl. Instrum. Methods Phys. Res. A.

[6] Kotov I.V. (2005). Currents induced by charges moving in semiconductor. Nucl. Instrum. Methods Phys. Res. A.

[7] Hamel L.A., Julien M. (2008). Generalized demonstration of Ramo’s theorem with space charge and polarization effects. Nucl. Instrum. Methods Phys. Res. A.

[8] Ramo S. (1939). Currents induced by electron motion. Proc. Inst. Radio Eng..

[9] Shockley A. (1938). Currents to conductors induced by a moving point charge. J. Appl. Phys..

[10] Korvink J.G., Greiner A. (2002). Semiconductors for Micro and Nanotechnology—An Introduction for Engineers.

[11] Blotekjer K. (1970). Transport equations for electrons in two-valley semiconductors. IEEE Trans. Electron. Devices.

[12] Sellin P.J., Vaitkus J. (2006). New materials for radiation hard semiconductor dectectors. Nucl. Instrum. Methods Phys. Res. A.

[13] Moll M. (2006). Radiation tolerant semiconductor sensors for tracking detectors. Nucl. Instrum. Methods Phys. Res. A.

[14] Gaubas E., Ceponis T., Jasiunas A., Kovalevskij V., Meskauskaite D., Pavlov J., Remeikis V., Tekorius A., Vaitkus J. (2014). Correlative analysis of the *in situ* changes of carrier decay and proton induced photoluminescence characteristics in chemical vapor deposition grown GaN. Appl. Phys. Lett..

[15] Pomorski M., Berdermann E., de Boer W., Furgeri A., Sander C., Morse J. (2007). Charge transport properties of single crystal CVD-diamond particle detectors. Diam. Relat. Mater..

[16] Nakhostin M. (2013). Charged particle response of transmission diamond detectors. Nucl. Instrum. Methods Phys. Res. A.

[17] Gaubas E., Ceponis T., Kalesinskas V. (2013). Currents induced by injected charge in junction detectors. Sensors.

[18] Gaubas E., Ceponis T., Pavlov J. (2015). Pulsed current signals in capacitor type particle detectors. J. Instrum..

[19] Gaubas E., Ceponis T., Pavlov J. (2015). Modelling of radiation damage recovery in particle detectors based on GaN. Nucl. Instrum. Methods Phys. Res. B.

[20] Gaubas E., Ceponis T., Pavlov J., Baskevicius A. (2014). Profiling of the injected charge drift current transients by cross-sectional scanning technique. J. Appl. Phys..

[21] Gaidukov G.N., Abramov A.A. (2008). An interpretation of the energy conservation law for a point charge moving in a uniform electric field. Phys. Usp..

[22] WolframAlpha Computational Knowledge Engine. http://www.wolframalpha.com.

[23] Dushek O., Kuzmin S. (2004). The fields of a moving point charge: A new derivation from Jefimenko’s equations. Eur. J. Phys..

[24] Jackson J.D. (1962). Classical Electrodynamics.

[25] Stratton J.A. (1941). Electromagnetic Theory.

[26] Griffiths D.J. (1999). Introduction to Electrodynamics.

[27] Di Bartolo B. (2004). Classical Theory of Electromagnetism.

[28] Gaubas E., Ceponis T., Vaitkus J., Raisanen J. (2011). Study of variations of the carrier recombination and charge transport parameters during proton irradiation of silicon pin diode structures. AIP Adv..

[29] Blood P., Orton J.W. (1992). The Electrical Characterization of Semiconductors: Majority Carriers and Electron States.

[30] Gaubas E., Vaitkus J., Simoen E., Claeys C., Vanhellemont J. (2001). Excess carrier cross-sectional technique for determination of the surface recombination velocity. Mater. Sci. Semicond. Process..

[31] Gaubas E. (2003). Transient absorption techniques for investigation of recombination properties in semiconductor materials. Lith. J. Phys..

[32] Luke K.L., Cheng L.J. (1987). Analysis of the interaction of a laser pulse with a silicon wafer: Determination of bulk lifetime and surface recombination velocity. J. Appl. Phys..

[33] Gaubas E., Vaitkus J., Smith K.M. (2011). Monitoring of carrier lifetime in GaAs substrate-epi-layer structures by space-resolved transient microwave absorption. Nucl. Instrum. Methods Phys. Res. A.

[34] Bonch-Bruyevich V.L., Kalashnikov S.G. (1977). Semiconductor Physics.

[35] Mott N.F., Gurney R.W. (1964). Electronic Processes in Ionic Crystals.

[36] Lampert M.A., Mark P. (1970). Current Injection in Solids.

[37] McIntyre R.J. (1966). Multiplication noise in uniform avalanche diodes. IEEE Trans. Electron. Devices.

[38] Baliga B.J. (2008). Fundamentals of Power Semiconductore Devices.

[39] Van Overstraeten R., de Man H. (1970). Measurement of the ionization rates in diffused silicon p-n junctions. Solid-State Electron..

[40] Maes W., de Meyer K., van Overstraeten R. (1990). Impact ionization in silicon: A review and update. Solid-State Electron..

[41] Spieler H. (2005). Semiconductor Detector Systems.

